# Comparative effectiveness of physical training modalities on swimming performance: a two-tier network meta-analysis

**DOI:** 10.3389/fphys.2025.1636595

**Published:** 2025-08-28

**Authors:** Ze Wang, Ke Liu, Xinming Zhao, Jie Gao

**Affiliations:** ^1^ School of Police Law Enforcement Abilities Training, People’s Public Security University of China, Beijing, China; ^2^ Department of Military Training, Officers College of PAP, Chengdu, China; ^3^ School of Education, Beijing Sport University, Beijing, China

**Keywords:** competitive swimming, physical training, swimming performance, network-meta analysis, randomized controlled trials

## Abstract

**Objective:**

To evaluate and compare the effectiveness of different physical training environments and modalities on swimming performance and sport-specific skills in competitive swimmers using a two-tier network meta-analysis.

**Methods:**

A systematic search of six databases identified 36 randomized controlled trials involving 844 competitive swimmers. A first-tier network meta-analysis compared aquatic, dry-land, and combined training environments across performance outcomes (25 m, 50 m, 100 m, 200 m times) and sport-specific metrics (start time, turn time, swim velocity, stroke rate, stroke length). A second-tier analysis further examined specific training modalities within combined and dry-land categories. Mean differences (MD) and standardized mean differences (SMD) with 95% confidence intervals (CI) were reported; interventions were ranked using surface under the cumulative ranking curve (SUCRA).

**Results:**

Combined training showed the highest efficacy across multiple outcomes. Compared to control, it significantly improved 100 m time (MD = −2.01 s; 95% CI: −2.87 to −1.16), swimming velocity (MD = 1.27 m/s; 95% CI: 0.61–1.94), stroke rate (SMD = 1.63; 95% CI: 0.92–2.34), and stroke length (SMD = 0.86; 95% CI: 0.23–1.49). In the second-tier analysis, water plus dry-land resistance training (W + DRT) ranked highest across 50 m, 100 m, swim velocity, and stroke metrics. Core training and power training showed specific benefits for 25 m sprint (MD = −0.90s; 95% CI: −1.79 to −0.01) and take-off velocity (MD = 0.18 m/s; 95% CI: 0.03–0.32).

**Conclusion:**

Combined aquatic and dry-land training—especially W + DRT—most effectively improves swimming performance and sport-specific skills. Core and power training function as targeted adjuncts. These findings provide a concise, precision-based prescription for physical preparation in competitive swimming.

## 1 Introduction

Competitive swimming is a highly technical and physically demanding sport that requires the integration of strength, power, speed, and endurance across a wide range of competitive distances (Price et al., 2024). While in-water technique and training remain central to performance development, growing evidence suggests that supplementary physical training beyond standard swimming routines is essential for optimizing athletic output. These components are critical to competitive success in both youth and adult swimmers (Price et al., 2024; Aspenes et al., 2009). Additional physical conditioning—targeting muscular strength, power, and neuromuscular control—has been shown to enhance key performance parameters such as swim velocity, stroke rate, stroke length, and explosive actions during starts and turns (Fone and van den Tillaar, 2022). These components are critical to competitive success, particularly in sprint events where minimal differences in power output and movement efficiency can determine outcomes (Zemková and Zapletalová, 2022). As such, structured physical training has become an indispensable component of comprehensive performance enhancement programs in both youth and competitive swimmers.

Resistance training (muscular contractions against external load) is widely used in swimming to build strength (Jin et al., 2024). Surveys of elite strength and conditioning coaches indicate that 90% of high-performance swim programs integrate both land- and water-based resistance modalities (Crowley et al., 2018). Such training may be performed in the water (e.g., using paddles, drag suits, tethers or swim flumes) or on land (e.g., weightlifting, plyometric jumps or swim-specific ergometry). Dry-land exercises—endorsed by national coaching guidelines—allow heavy loading to target maximal strength and power, whereas aquatic drills provide high specificity and reduced musculoskeletal impact (Morou et al., 2012). High-intensity interval training (HIIT) may be applied either in the pool (short sprint repeats) or on land (cycling or running intervals) to improve cardiovascular and metabolic conditioning. Core stability and neuromuscular exercises (using mats, Swiss balls or other devices) are also common to enhance trunk control and streamline position. Coaches often combine modalities; indeed, a recent review found that combined swimming-plus-strength training regimens produced larger performance gains than swim-only training (Fone and van den Tillaar, 2022), suggesting an additive benefit of integrating aquatic and dry-land approaches.

Several systematic reviews and meta-analyses have examined targeted training interventions in swimmers. For example, a recent meta-analysis reported that resistance training significantly enhanced swimmers’ upper-body strength and front-crawl performance (likely via increased stroke rate) (Jin et al., 2024). Another meta-analysis found that plyometric jump training in water sports significantly improved swimmers’ physical fitness and sport-specific outputs (such as starts and turns) compared to conventional training (Ramirez-Campillo et al., 2022). A systematic review of strength-training methods in swimming concluded that all intervention types yielded modest gains (2%–2.5%) in race performance (Lum and Barbosa, 2019) with combined swim-plus-strength approaches tending to show slightly greater effects. However, these analyses mainly compared each modality to control or usual training and did not directly contrast multiple modalities with each other. As a result, there is no clear consensus on the relative efficacy of aquatic versus dry-land versus combined training approaches. This gap highlights the need for an integrative analysis of all modalities.

Network meta-analysis (NMA) provides a rigorous and comprehensive statistical approach to simultaneously compare multiple interventions by synthesizing both direct and indirect evidence, thereby yielding coherent estimates across an entire treatment network and allowing probabilistic ranking of comparative efficacy (e.g., SUCRA) (Salanti, 2012; Rücker and Schwarzer, 2014). Unlike traditional pairwise meta-analysis, NMA enables the estimation of relative effectiveness across a full set of competing interventions within a connected evidence structure. When intervention strategies differ along more than one dimension (e.g., training environments and specific training modalities), a hierarchical or “two-tier” NMA framework is particularly relevant to preserve clinical coherence and interpretability (Welton et al., 2009; Nikolakopoulou et al., 2014). Therefore, the present study employed a two-level NMA to systematically evaluate the effectiveness of physical training strategies for swimmers. In the first stage, we examined the effects of different training environments, including aquatic, dry-land, and combined approaches, on swimming performance and sport-specific outcomes. Based on the most effective training environment identified in the first-tier analysis, a second-tier NMA was conducted to further compare the effectiveness of specific training modalities, such as strength training, plyometric training, and core training. This hierarchical approach provides more detailed and clinically applicable evidence for optimizing training prescriptions in competitive swimming.

## 2 Methods

This systematic review and NMA was reported according to the Preferred Reporting Items for Systematic Reviews and Meta-Analyses (PRISMA) 2020 statement and its extension for network meta-analyses (PRISMA-NMA) (Hutton et al., 2015; Page et al., 2021). Ethical approval and consent statements were not required as this study synthesizes previously published data. The protocol was prospectively registered (CRD420251059608).

### 2.1 Data sources and search strategy

A systematic literature search was conducted across PubMed, Medline, Embase, PsycINFO, Cochrane Central Register of Controlled Trials (CENTRAL), and Web of Science from database inception to 18 May 2025. The search strategy included key terms such as “swimming,” “athletes,” “training,” “strength exercise,” and “sports performance.” The full search strategy, detailing exact terms and combinations, is provided in Supplementary Material S1. Reference lists from included studies and bibliographies of relevant systematic reviews published within the past 5 years were manually reviewed to identify additional eligible studies. Two independent reviewers screened titles, abstracts, and full texts, with discrepancies resolved through discussion or arbitration by a third reviewer.

### 2.2 Study selection

Studies were included if they met the following criteria (Price et al., 2024): participants were competitive swimmers (e.g., professional, amateur, or youth swimmers) (Aspenes et al., 2009); interventions included any type of physical training aimed at improving swimming performance (Fone and van den Tillaar, 2022); control conditions included no intervention, regular swimming training, or alternative physical training modalities for head-to-head comparisons in the network meta-analysis (Zemková and Zapletalová, 2022); reported at least one performance-related outcome such as race performance or sport-specific skills; and (Jin et al., 2024) randomized controlled trial (RCT) design.

Studies were excluded if they (Price et al., 2024): recruited non-swimming athletes or special populations (e.g., hearing-impaired, disabled swimmers) (Aspenes et al., 2009); evaluated acute effects of training (Fone and van den Tillaar, 2022); combined physical training with non-training interventions (e.g., electrical stimulation, nutritional supplementation) (Zemková and Zapletalová, 2022); lacked clear description of training types; or (Jin et al., 2024) did not report means and standard deviations (SD), and data were unobtainable after repeated attempts at contacting authors. Two reviewers independently assessed eligibility based on predefined criteria through examination of titles, abstracts, and full texts.

### 2.3 Data extraction

Eligible studies were managed using EndNote X9 software. Two reviewers independently extracted relevant publication details (authors, title, year, journal), participant characteristics (sample size, age, gender), intervention specifics (training content, intensity, duration, frequency, and intervention period), and outcome measures (Supplementary Material S3). Change scores (endpoint minus baseline values), SDs, and sample sizes were extracted for effect size calculations. Missing mean changes and SDs were derived following guidelines from the Cochrane Handbook (Higgins and Green, 2008). Where necessary, authors were contacted at least four times over 6 weeks to obtain missing data.

### 2.4 Outcomes

Primary outcomes included swimming performance and sport-specific skills. Swimming performance was assessed via competitive times over standard distances (25 m, 50 m, 100 m, 200 m). Sport-specific skills included start speed, turn time, start reaction time, swimming velocity, stroke rate, and stroke length, measured through standardized timing systems, digital analysis, or biomechanical evaluations.

### 2.5 Risk of bias assessment

Risk of bias was evaluated at the study level using the revised Cochrane risk-of-bias tool (RoB 2) (Sterne et al., 2019), which addresses domains including randomization process, deviations from intended interventions, missing outcome data, measurement of outcomes, and selection of reported results. Discrepancies were resolved through consultation with a third reviewer to ensure rigorous, unbiased evaluation of included studies.

### 2.6 Data coding

A two-tiered network meta-analysis approach was employed. Initially, studies were categorized into aquatic training, dry-land training, combined aquatic and dry-land training, and control groups (CON). Aquatic training (ART, AST) was defined as supplemental in-water interventions—such as tethered swimming, drag suit drills, paddle exercises, or swim-flume sessions—explicitly administered in addition to athletes’ regular pool workouts, thereby excluding routine swim-only training. Dry-land training (DRT) encompassed land-based modalities—including resistance exercises, plyometric drills, high-intensity interval training, core stability work, and power development activities—delivered alongside usual swim practice to augment performance. Combined training referred to the concurrent application of both aquatic and dry-land modalities, while control groups continued regular swim training or received no additional intervention. Subsequently, for a more detailed second-tier analysis, aquatic training was divided into aquatic resistance training (ART) and aquatic speed training (AST); dry-land training was further subdivided into core training (CT), high-intensity interval training (HIIT), plyometric jump training (PJT), power training (PT), and resistance training (RT); and combined training was separated into combined aquatic HIIT and land resistance training (A-HIIT + L-RT) and combined water and dry-land resistance training (W + DRT).

### 2.7 Data analysis

Data were analyzed using Stata version 17.0 (StataCorp LLC, Texas, United States). Network meta-analyses compared the effects of different training modalities on swimming performance and specific skills. Initially, a first-tier NMA assessed the comparative effectiveness among aquatic, dry-land, and combined training modalities. Based on these results, a second-tier NMA was conducted to examine the efficacy of distinct training approaches within the most effective category identified in the first-tier analysis. Network plots were generated to illustrate the comparative connections among interventions.

Considering anticipated clinical heterogeneity, random-effects models were utilized to accommodate within- and between-study variability. Due to methodological and measurement differences in stroke rate and stroke length, standardized mean differences (SMD) with 95% confidence intervals (CIs) were used; all other outcomes were expressed as mean differences (MD) with 95% CIs. Heterogeneity was evaluated using the I^2^ statistic, categorized as low (25%), moderate (50%), or high (75%). Bayesian frameworks implemented through Stata packages ‘network’ and ‘mvmeta’ facilitated the network meta-analysis.

Interventions were ranked according to the Surface Under the Cumulative Ranking curve (SUCRA) values, with higher SUCRA indicating greater relative effectiveness. Adjusted funnel plots and Egger’s test assessed publication bias, with a p-value <0.05 indicating potential bias (Chaimani et al., 2013). Prediction interval plots were also generated to further explore heterogeneity and variability in effect sizes. All statistical tests were two-sided, and statistical significance was set at p < 0.05.

## 3 Results

### 3.1 Characteristics of included studies

**FIGURE 1:**
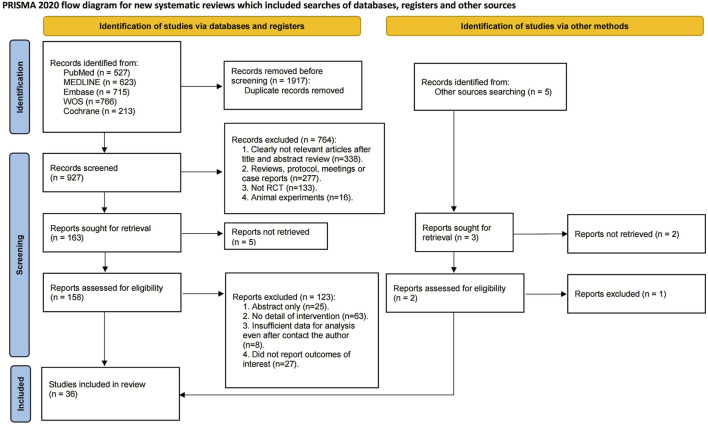
PRISMA Flow diagram of the search process for studies.

A total of 2,844 records were initially identified through electronic database searches. After removing 1917 duplicates, 927 records underwent title and abstract screening, resulting in the exclusion of 764 articles. Subsequently, 158 articles were assessed for full-text eligibility, ultimately leading to the inclusion of 36 randomized controlled trials (RCTs), involving 844 swimmers in the systematic review and network meta-analysis (Figure 1) (Aspenes et al., 2009; Amara et al., 2022; Amara et al., 2021; Amara et al., 2023; Amaro et al., 2017; Aouani et al., 2024; Bishop et al., 2009; Born et al., 2020; Breed and Young, 2003; Caas et al., 2020; Chortane et al., 2022; Cossor et al., 1999; Czuba et al., 2017; Dragunas et al., 2012; Garrido et al., 2010; YGJAJoE and Training, 2018; Girold et al., 2006; Girold et al., 2012; Girold et al., 2007; Gourgoulis et al., 2019; Jones et al., 2017; Karpiński et al., 2020; Khiyami et al., 2022; Kilen et al., 2014; Lopes et al., 2021; Naczk et al., 2017; Norberto et al., 2023; Nugent et al., 2019; Patil et al., 2014; Potdevin et al., 2011; Sadowski et al., 2020; Sammoud et al., 2021; Sammoud et al., 2019; Sperlich et al., 2010; Toussaint and Vervoorn, 1990; Weston et al., 2015). Detailed characteristics of these studies are presented in Supplementary Material 2.

The included studies were published between 1990 and 2024, with a median publication year of 2017. Sample sizes ranged from 12 to 60 participants per study, with a median of 22 swimmers. Among the studies, three exclusively involved female participants, while 14 exclusively involved males. Because most trials did not report separate outcome data by sex, we were unable to perform sex-specific analyses; thus, pooled effect estimates represent mixed-sex cohorts without adjustment for potential sex differences. Participants’ ages ranged from 10.0 to 21.4 years, with a median age of 16.2 years. Thirty-four studies provided detailed anthropometric data, with participants’ average heights ranging from 140 cm to 183 cm (median 171 cm), and average body weights ranging from 36.2 kg to 78.9 kg (median 65.4 kg).

### 3.2 Results of network meta-analysis

#### 3.2.1 First-tier network meta-analysis

##### 3.2.1.1 Swimming performance

**FIGURE 2:**
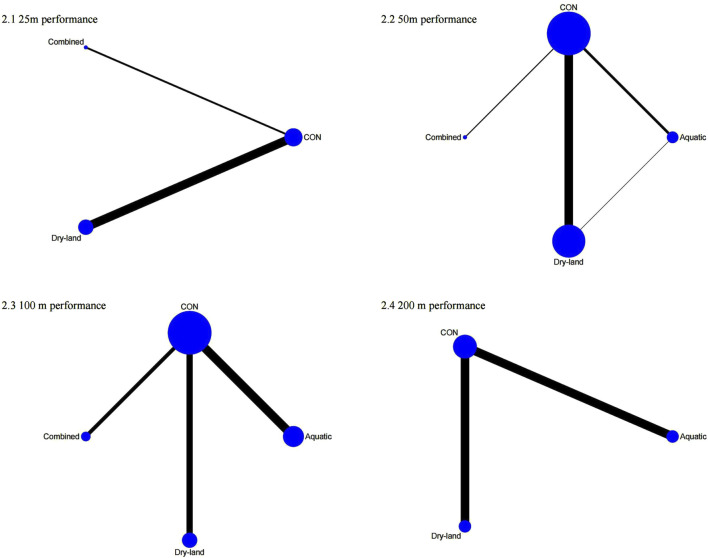
Network plots of first-tier network meta-analysis for swimming performance. 1: 25 m performance; 2: 50 m performance; 3: 100 m performance; 4: 200 m performance.

**FIGURE 3:**
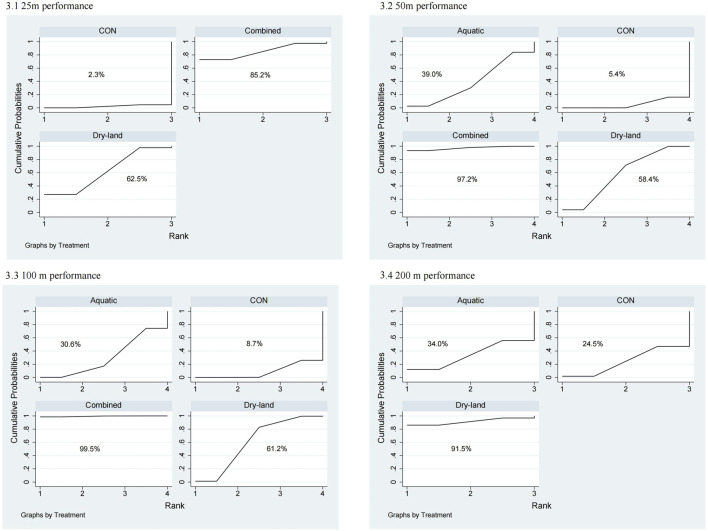
SUCRA rankings from first-tier network meta-analysis for swimming performance. 3.1: 25 m performance; 3.2: 50 m performance; 3.3: 100 m performance; 3.4: 200 m performance.

The first-tier network meta-analysis for 25 m performance included six studies with 140 swimmers Figure 2. As shown in Figure 3, combined training ranked highest according to the SUCRA rankings (85.2%), followed by dry-land training (62.5%). Compared to the control (CON) group, combined training (MD = −0.90, 95% CI: 1.77 to −0.03) and dry-land training (MD = −0.57, 95% CI: 1.11 to −0.02) significantly improved 25m performance (Table 1).

**TABLE 1:** League table of the first-level network meta-analysis results.

1.1 25 m performance		
Combined		
−0.33 (−1.36,0.69)	Dry-land	
**−0.90 (-1.77,-0.03)**	**−0.57 (-1.11,-0.02)**	CON

1.2 50 m performance			
Combined			
−0.60 (−1.30,0.10)	Dry-land		
−0.76 (−1.58,0.06)	−0.16 (−0.73,0.41)	Aquatic	
**−1.01 (-1.66,-0.37)**	**−0.41 (-0.68,-0.14)**	−0.25 (−0.75,0.25)	CON

100 m performance			
**−1.22 (-2.28,-0.16)**	Dry-land		
**−1.72 (-2.92,-0.53)**	−0.50 (−1.54,0.53)	Aquatic	
**−2.01 (-2.87,-1.16)**	**−0.79 (-1.41,-0.17)**	−0.29 (−1.12,0.54)	CON

200 m performance		
Dry-land		
−39.98 (−109.58,29.62)	Aquatic	
−43.10 (−92.71,6.51)	−3.12 (−51.94,45.71)	CON

Start time		
Combined		
−0.05 (−0.15,0.06)	Dry-land	
−0.09 (−0.18,-0.00)	−0.04 (−0.10,0.02)	CON

Turn time		
Combined		
**−0.25 (-0.49,-0.02)**	Dry-land	
**−0.27 (-0.42,-0.12)**	−0.02 (−0.19,0.16)	CON

Swimming velocity			
Turn time			
**1.01 (0.25,1.77)**	Dry-land		
**1.23 (0.36,2.10)**	0.22 (−0.46,0.89)	Aquatic	
**1.27 (0.61,1.94)**	0.26 (−0.10,0.63)	0.05 (−0.52,0.61)	CON

Stroke rate			
Combined			
**1.38 (0.58,2.18)**	Aquatic		
**1.57 (0.79,2.36)**	0.20 (−0.28,0.67)	Dry-land	
**1.63 (0.92,2.34)**	0.25 (−0.11,0.62)	0.06 (−0.27,0.39)	CON

Stroke length			
Combined			
0.64 (−0.07,1.36)	Dry-land		
**0.83 (0.09,1.56)**	0.19 (−0.30,0.67)	Aquatic	
**0.86 (0.23,1.49)**	0.21 (−0.12,0.55)	0.03 (−0.35,0.40)	CON

Bold values indicate statistically significant differences (p < 0.05).

For 50m performance, 27 studies involving 570 swimmers were analyzed Figure 3. Combined training demonstrated the highest efficacy (SUCRA = 97.2%), followed by dry-land training (SUCRA = 58.4%). Both combined (MD = −1.01, 95% CI: 1.66 to −0.37) and dry-land training (MD = −0.41, 95% CI: 0.68 to −0.14) significantly reduced 50m swim times compared to CON (Table 1).

In the 100 m performance analysis (15 studies, 317 swimmers), combined training was ranked highest (SUCRA = 99.5%), significantly outperforming dry-land (MD = −1.22, 95% CI: 2.28 to −0.16), aquatic (MD = −1.72, 95% CI: 2.92 to −0.53), and CON training (MD = −2.01, 95% CI: 2.87 to −1.16). Dry-land training also significantly improved performance compared to CON (MD = −0.79, 95% CI: 1.41 to −0.17; Table 1).

The 200 m performance analysis (8 studies, 155 swimmers) revealed combined training as optimal (SUCRA = 91.5%), followed by aquatic training (SUCRA = 34.0%). However, no significant differences were detected among interventions (Table 1).

##### 3.2.1.2 Sport-specific skills

**FIGURE 4:**
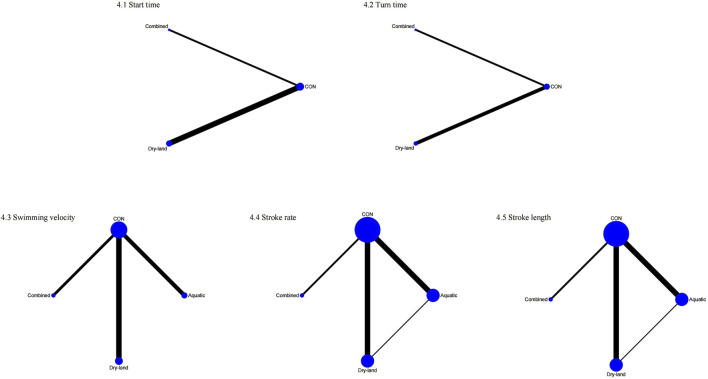
Network plots of first-tier network meta-analysis for sport-specific skills. 1: Start time; 2: Turn time; 3: Swimming velocity; 4: Stroke rate; 5: Stroke length.

**FIGURE 5:**
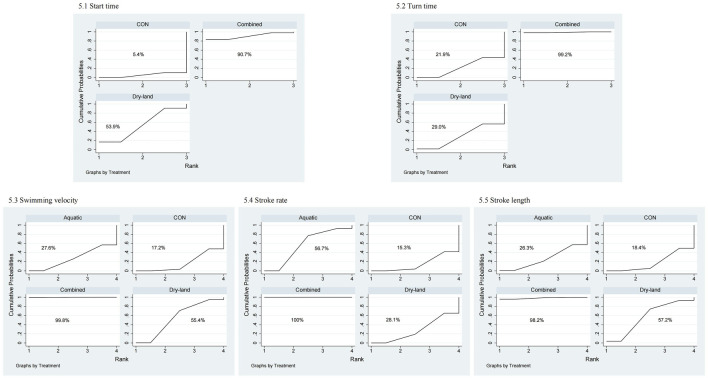
SUCRA rankings from first-tier network meta-analysis for sport-specific skills. 1: Start time; 2: Turn time; 3: Swimming velocity; 4: Stroke rate; 5: Stroke length.

For start time, four studies with 98 swimmers indicated combined training as the optimal method (SUCRA = 90.7%), followed by dry-land (SUCRA = 53.9%; Figures 4, 5). No statistically significant differences were found among the interventions (Table 1).

Three studies involving 76 swimmers evaluated turn time. Combined training was optimal (SUCRA = 99.2%), significantly outperforming dry-land (MD = −0.25, 95% CI: 0.49 to −0.02) and CON (MD = −0.27, 95% CI: 0.42 to −0.12; Table 1).

Swimming velocity was assessed in nine studies (210 swimmers), identifying combined training as most effective (SUCRA = 99.8%). Combined training significantly improved velocity compared to dry-land (MD = 1.01, 95% CI: 0.25–1.77), aquatic (MD = 1.23, 95% CI: 0.36–2.10), and CON (MD = 1.27, 95% CI: 0.61 to 1.94; Table 1).

Stroke rate was analyzed in 13 studies (291 swimmers), with combined training (SUCRA = 100%) significantly outperforming aquatic (SMD = 1.38, 95% CI: 0.58–2.18), dry-land (SMD = 1.57, 95% CI: 0.79–2.36), and CON groups (SMD = 1.63, 95% CI: 0.92 to 2.34; Table 1).

Stroke length was evaluated across 13 studies (284 swimmers), showing combined training as optimal (SUCRA = 98.2%). Combined training significantly improved stroke length compared to aquatic (SMD = 0.83, 95% CI: 0.09–1.56) and CON (SMD = 0.86, 95% CI: 0.23 to 1.49; Table 1).

#### 3.2.2 Second-tier network meta-analysis

Based on the results of the first-tier network meta-analysis, combined training consistently ranked as the most effective intervention for improving both swimming performance and sport-specific skills, with dry-land training frequently ranking second. Furthermore, dry-land training demonstrated statistically significant improvements over control in several performance indicators (e.g., 25 m, 50m, and 100 m swim times). Therefore, a second-tier network meta-analysis was conducted to further evaluate the comparative effectiveness of specific training modalities within the combined and dry-land domains.

##### 3.2.2.1 Swimming performance

**FIGURE 6:**
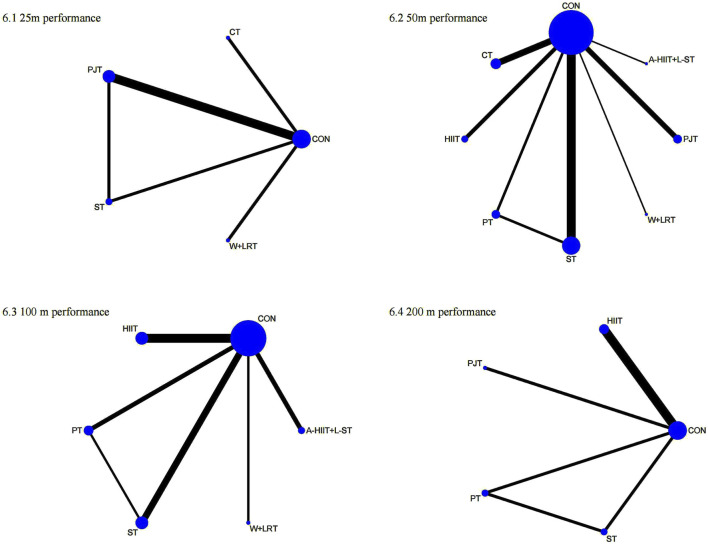
Network plots of second-tier network meta-analysis for swimming performance. 1: 25 m performance; 2: 50 m performance; 3: 100 m performance; 4: 200 m performance.

**FIGURE 7:**
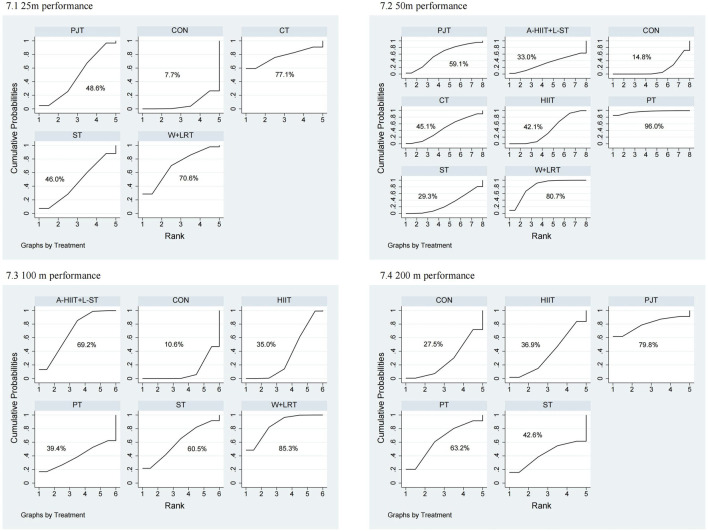
SUCRA rankings from second-tier network meta-analysis for swimming performance. 1: 25 m performance; 2: 50 m performance; 3: 100 m performance; 4: 200 m performance.

As shown in Figure 6 the second-tier network meta-analysis for 25 m performance included 7 studies with a total of 161 swimmers, evaluating different formats of combined and dry-land training. As illustrated in Figure 7 (7.1), core training (CT) demonstrated the highest probability of being the most effective intervention (SUCRA = 77.1%), followed by combined water and dry-land resistance training (W + DRT, SUCRA = 70.6%). As shown in Table 2, W + DRT significantly reduced 25 m swim time compared to the control group (MD = −0.90, 95% CI: 1.79 to −0.01).

**TABLE 2:** League table of the second-level network meta-analysis results.

25 m performance				
CT				
−0.36 (−2.45,1.73)	W + DRT			
−0.75 (−2.73,1.23)	−0.39 (−1.47,0.69)	PJT		
−0.77 (−2.85,1.31)	−0.41 (−1.66,0.83)	−0.02 (−0.87,0.83)	RT	
−1.26 (−3.15,0.63)	**−0.90 (-1.79,-0.01)**	−0.51 (−1.11,0.09)	−0.49 (−1.36,0.38)	CON

50 m performance							
PT							
−1.26 (−3.35,0.83)	W + DRT						
−1.73 (−3.94,0.49)	−0.46 (−1.70,0.77)	PJT					
−2.01 (−4.17,0.15)	−0.75 (−1.88,0.38)	−0.29 (−1.63,1.06)	CT				
**−2.11 (-4.10,-0.12)**	**−0.85 (-1.61,-0.08)**	−0.38 (−1.44,0.67)	−0.10 (−1.03,0.83)	HIIT			
−2.30 (−4.84,0.24)	−1.04 (−2.79,0.71)	−0.58 (−2.47,1.32)	−0.29 (−2.12,1.54)	−0.19 (−1.82,1.44)	A-HIIT + L-RT		
**−2.28 (-4.20,-0.35)**	**−1.02 (-2.01,-0.02)**	−0.55 (−1.79,0.68)	−0.27 (−1.40,0.87)	−0.17 (−0.93,0.60)	0.02 (−1.73,1.77)	RT	
**−2.44 (-4.41,-0.47)**	**−1.18 (-1.89,-0.47)**	−0.72 (−1.73,0.29)	−0.43 (−1.31,0.45)	**−0.33 (-0.62,-0.04)**	−0.14 (−1.74,1.46)	−0.16 (−0.87,0.54)	CON

100 m performance					
W + DRT					
−0.61 (−2.34,1.11)	A-HIIT + L-RT				
−0.83 (−3.64,1.99)	−0.21 (−2.98,2.55)	RT			
−1.80 (−5.99,2.40)	−1.18 (−5.34,2.98)	−0.97 (−5.65,3.71)	PT		
**−1.80 (-3.18,-0.42)**	−1.19 (−2.46,0.08)	−0.98 (−3.54,1.59)	−0.01 (−4.04,4.02)	HIIT	
**−2.35 (-3.63,-1.07)**	**−1.74 (-2.89,-0.58)**	−1.52 (−4.04,0.99)	−0.55 (−4.55,3.44)	**−0.55 (-1.07,-0.03)**	CON

200 m performance				
PJT				
−8.10 (−38.76,22.56)	PT			
−12.70 (−49.06,23.66)	−4.60 (−29.29,20.09)	RT		
−14.49 (−40.50,11.52)	−6.39 (−23.13,10.34)	−1.79 (−27.53,23.94)	HIIT	
−15.10 (−40.95,10.75)	−7.00 (−23.48,9.48)	−2.40 (−27.97,23.17)	−0.61 (−3.50,2.28)	CON

CT			
Take-off velocity			
0.85 (−1.41,3.11)	PJT		
0.85 (−1.42,3.13)	0.00 (−0.28,0.28)	RT	
1.03 (−1.23,3.29)	**0.18 (0.03,0.32)**	0.18 (−0.14,0.49)	CON

Start time			
W + DRT			
−0.04 (−0.28,0.20)	CT		
−0.05 (−0.27,0.16)	−0.01 (−0.23,0.20)	PJT	
−0.09 (−0.26,0.08)	−0.05 (−0.22,0.12)	−0.04 (−0.17,0.10)	CON

Turn time			
W + DRT			
−0.24 (−2.68,2.20)	CT		
−0.27 (−2.05,1.51)	−0.03 (−1.69,1.63)	CON	
−0.38 (−3.48,2.72)	−0.14 (−3.17,2.89)	−0.11 (−2.64,2.42)	PJT

Swimming velocity					
W + DRT					
0.02 (−1.30,1.35)	A-HIIT + L-RT				
0.80 (−0.46,2.05)	0.77 (−0.48,2.02)	PJT			
**1.08 (0.05,2.10)**	**1.05 (0.04,2.07)**	0.28 (−0.65,1.21)	CT		
**1.29 (0.35,2.23)**	**1.26 (0.33,2.19)**	0.49 (−0.34,1.32)	0.21 (−0.20,0.62)	CON	
**1.82 (0.42,3.22)**	**1.80 (0.40,3.19)**	1.02 (−0.31,2.36)	0.74 (−0.37,1.86)	0.53 (−0.51,1.58)	HIIT

Stroke rate					
W + DRT					
0.67 (−0.76,2.09)	A-HIIT + L-RT				
**1.55 (0.31,2.78)**	0.88 (−0.24,2.00)	RT			
**1.64 (0.37,2.90)**	0.97 (−0.19,2.13)	0.09 (−0.55,0.73)	PT		
**2.01 (0.94,3.08)**	**1.34 (0.40,2.28)**	0.46 (−0.15,1.07)	0.37 (−0.30,1.04)	CON	
**2.08 (0.93,3.22)**	**1.41 (0.38,2.43)**	0.53 (−0.21,1.26)	0.44 (−0.35,1.22)	0.07 (−0.34,0.47)	CT

Stroke length						
W + DRT						
0.78 (−1.74,3.30)	A-HIIT + L-RT					
0.95 (−1.22,3.11)	0.17 (−1.96,2.29)	RT				
1.02 (−1.55,3.60)	0.24 (−2.30,2.79)	0.08 (−2.12,2.28)	HIIT			
1.01 (−1.25,3.28)	0.23 (−2.00,2.46)	0.07 (−1.30,1.43)	−0.01 (−2.31,2.29)	PT		
1.21 (−0.85,3.28)	0.44 (−1.59,2.46)	0.27 (−1.30,1.83)	0.19 (−1.91,2.29)	0.20 (−1.50,1.91)	CT	
1.29 (−0.52,3.09)	0.51 (−1.25,2.26)	0.34 (−0.86,1.54)	0.26 (−1.58,2.11)	0.27 (−1.10,1.65)	0.07 (−0.94,1.08)	CON

Bold values indicate statistically significant differences (p < 0.05).

For 50m performance, 21 studies with 481 swimmers were analyzed Figure 7 (7.2). Power training (PT) ranked highest (SUCRA = 96.0%), followed by W + DRT (SUCRA = 80.7%). Table 2 shows that PT significantly improved performance compared to HIIT (MD = −2.11, 95% CI: 4.10 to −0.12), RT (MD = −2.28, 95% CI: 4.20 to −0.35), and CON (MD = −2.44, 95% CI: 4.41 to −0.47). W + DRT also significantly outperformed HIIT (MD = −0.85, 95% CI: 1.61 to −0.08), RT (MD = −1.02, 95% CI: 2.01 to −0.02), and CON (MD = −1.18, 95% CI: 1.89 to −0.47). In addition, HIIT showed a moderate but significant benefit over CON (MD = −0.33, 95% CI: 0.62 to −0.04).

The 100 m analysis included 11 studies with 250 swimmers Figure 7 (7.3). W + DRT ranked highest (SUCRA = 85.3%), followed by combined aquatic HIIT and land strength training (A-HIIT + L-RT, SUCRA = 69.2%). As shown in Table 2, W + DRT significantly outperformed HIIT (MD = −1.80, 95% CI: 3.18 to −0.42) and CON (MD = −2.35, 95% CI: 3.63 to −1.07). A-HIIT + L-RT also significantly improved performance compared to CON (MD = −1.74, 95% CI: 2.89 to −0.58), and HIIT significantly outperformed CON (MD = −0.55, 95% CI: 1.07 to −0.03).

In the 200m event, five studies comprising 114 swimmers were included (Figure 6). Figure 7 (7.4) PJT showed the highest SUCRA ranking (79.8%), followed by PT (63.2%). However, Table 2 indicates that no statistically significant differences were observed between interventions.

##### 3.2.2.2 Sport-specific skills

**FIGURE 8:**
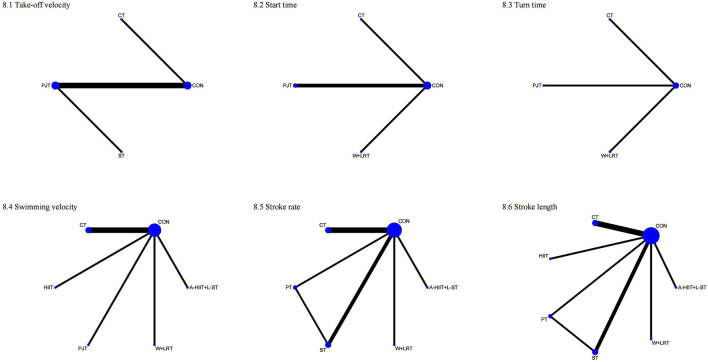
Network plots of second-tier network meta-analysis for sport-specific skills. 1: Take-off velocity; 2: Start time; 3: Turn time; 4: Swimming velocity; 5: Stroke rate; 6: Stroke length.

**FIGURE 9:**
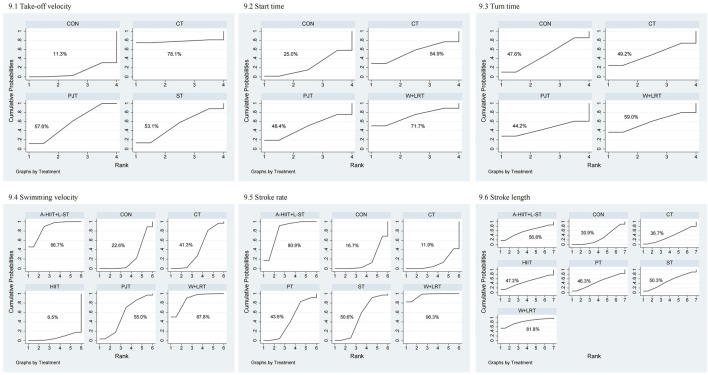
SUCRA rankings from second-tier network meta-analysis for sport-specific skills. 1: Take-off velocity; 2: Start time; 3: Turn time; 4: Swimming velocity; 5: Stroke rate; 6: Stroke length.

As shown in Figure 8 the second-tier meta-analysis for take-off velocity included five studies with 120 swimmers. As shown in Figure 9 (9.1), CT ranked highest (SUCRA = 78.1%), followed by PJT (SUCRA = 57.6%). Table 2 shows that PJT significantly improved take-off velocity compared to CON (MD = 0.18, 95% CI: 0.03–0.32).

In the start time analysis (4 studies, 98 swimmers), W + DRT ranked highest (SUCRA = 71.7%), followed by CT (SUCRA = 54.9%; Figure 9 (9.2)). Table 2 shows no significant differences between interventions.

For turn time (3 studies, 76 swimmers), W + DRT was optimal (SUCRA = 59.0%), followed by CT (SUCRA = 49.2%; Figure 9 (9.3)). No statistically significant differences were identified (Table 2).

Swimming velocity was evaluated in 7 studies with 176 swimmers Figure 9 (9.4). W + DRT ranked highest (SUCRA = 87.8%), followed closely by A-HIIT + L-RT (SUCRA = 86.7%). W + DRT significantly outperformed CT (MD = 1.08, 95% CI: 0.05–2.10), CON (MD = 1.29, 95% CI: 0.35–2.23), and HIIT (MD = 1.82, 95% CI: 0.42–3.22). A-HIIT + L-RT also demonstrated superiority over CT (MD = 1.05, 95% CI: 0.04–2.07), CON (MD = 1.26, 95% CI: 0.33–2.19), and HIIT (MD = 1.80, 95% CI: 0.40 to 3.19; Table 2).

Stroke rate was examined in 9 studies with 216 swimmers Figure 9 (9.5). W + DRT ranked highest (SUCRA = 96.3%), followed by A-HIIT + L-RT (SUCRA = 80.9%). As shown in Table 2, W + DRT significantly improved stroke rate compared to RT (SMD = 1.55, 95% CI: 0.31–2.78), PT (SMD = 1.64, 95% CI: 0.37–2.90), CON (SMD = 2.01, 95% CI: 0.94–3.08), and CT (SMD = 2.08, 95% CI: 0.93–3.22). A-HIIT + L-RT also showed significant improvements over CON (SMD = 1.34, 95% CI: 0.40–2.28) and CT (SMD = 1.41, 95% CI: 0.38–2.43).

In the stroke length analysis (10 studies, 231 swimmers), W + DRT ranked highest (SUCRA = 81.8%), followed by A-HIIT + L-RT (SUCRA = 56.8%; Figure 9 (9.6)). However, Table 2 indicates no statistically significant differences between the interventions.

### 3.3 Risk of bias and publication bias

Among the 36 included trials, 30 were assessed as having a low overall risk of bias, 5 were judged to have some concerns, and 1 study was rated as having a high risk. For the randomization process, 34 trials were at low risk, 1 showed some concerns, and 1 had high risk. All studies were rated as low risk for deviations from intended interventions. Regarding missing outcome data, 35 studies were considered low risk, and 1 was rated high risk. All studies were judged to have low risk in the domain of outcome measurement (Supplementary Material S3).

Potential publication bias was assessed using funnel plots (Supplementary Material S4, S5). Scatter plots showed varying degrees of symmetry, suggesting possible bias. Specifically, Figure 5 exhibited relatively balanced distributions, whereas other plots showed asymmetry indicative of potential bias. However, Egger’s test results were all non-significant (p > 0.05), indicating no substantial evidence of publication bias across the included studies.

## 4 Discussion

This network meta-analysis integrated data from 36 randomized controlled trials involving 844 competitive swimmers to compare the effects of different physical training environments and modalities on swimming performance and sport-specific skills. Three key findings emerged. First, in the first-tier analysis, combined aquatic and dry-land training consistently ranked highest across multiple performance outcomes—including short-distance swim times, swimming velocity, stroke rate, and stroke length—highlighting its multidimensional benefits and supporting its use as the most evidence-based strategy for enhancing competitive swimming performance. This pattern is congruent with prior systematic reviews and meta-analyses showing that swim-plus-resistance or combined programs outperform swim-only or single-modality approaches (Fone and van den Tillaar, 2022; Jin et al., 2024; Ramirez-Campillo et al., 2022; Guo et al., 2022; Muniz-Pardos et al., 2022). Second, in the second-tier analysis, W + DRT demonstrated superior efficacy across several key indicators, including 50m and 100 m times, swimming velocity, stroke rate, and stroke length, suggesting its particular effectiveness in improving power output, technical control, and movement efficiency. To our knowledge, no previous review has ranked combined submodalities head-to-head; our findings therefore provide the first evidence-based hierarchy of these approaches (Jin et al., 2024; Guo et al., 2022; Ruiz-Navarro et al., 2025). Third, within dry-land modalities, core training and power training showed targeted benefits, notably in improving 25 m performance and take-off velocity. Recent meta-analyses and randomized trials corroborate these modality-specific advantages for explosive actions and trunk control (Ramirez-Campillo et al., 2022; Khiyami et al., 2022; Rodríguez et al., 2025). While not as broadly effective as W + DRT, these approaches may serve as valuable components of individualized training programs tailored to specific athlete needs and event demands.

A key rationale for conducting the first-tier network meta-analysis was to clarify the relative efficacy of physical training across different environments—a question of particular relevance given previous concerns that dry-land strength and conditioning may not always translate into meaningful improvements in swimming performance (Crowley et al., 2018). Traditional training programs often rely heavily on land-based modalities; however, due to the biomechanical and neuromuscular specificity required in aquatic environments, the transferability of these gains has remained uncertain (Hermosilla et al., 2021). By synthesizing both direct and indirect evidence, the present analysis demonstrated that combined aquatic and dry-land training consistently produced the greatest improvements across 25 m, 50 m, and 100 m times, swimming velocity, stroke rate, and stroke length, thereby reinforcing a hybrid model that leverages environment-specific adaptations (Fone and van den Tillaar, 2022; Jin et al., 2024; Ramirez-Campillo et al., 2022; Guo et al., 2022; Muniz-Pardos et al., 2022; Ruiz-Navarro et al., 2025; Rodríguez et al., 2025). These findings provide robust empirical support for a hybrid training approach that integrates both environments to maximize training efficacy. This conclusion aligns partially with prior systematic reviews, which suggested that swim-plus-strength protocols may yield marginally greater performance benefits than swim-only or strength-only programs (Fone and van den Tillaar, 2022; Muniz-Pardos et al., 2019). However, most earlier studies were limited to pairwise comparisons and lacked the statistical power to establish a hierarchy of interventions. Our findings extend this body of evidence by demonstrating that the combination of aquatic and land-based training consistently outperforms isolated modalities across multiple domains of performance.

From a mechanistic standpoint, the superiority of combined training may stem from its ability to simultaneously target central physiological systems and movement-specific adaptations. Land-based resistance and power training provide high external loads and neuromuscular overload essential for developing maximal force output and rate of force development—qualities essential for sprint swimming (Marques et al., 2020). Conversely, aquatic resistance or technique-focused drills enhance proprioception, technical precision, and stroke mechanics under fluid-dynamic conditions, facilitating context-specific motor learning (Amara et al., 2022; Coloretti et al., 2025; Takagi et al., 2023). The integration of these two environments likely promotes enhanced intermuscular coordination, greater movement economy, and context-specific neuromuscular adaptations, thereby bridging the gap between general physical capacity and sport-specific performance (Tomazin et al., 2022). Conversely, aquatic-only training—though specific in movement patterns—may lack sufficient loading stimulus to elicit substantial neuromuscular or strength gains, particularly in already-trained swimmers. This may explain the relatively lower efficacy observed for aquatic training alone in our analysis (Ruiz-Navarro et al., 2025; Cortesi et al., 2024).

Building on the first-tier analysis, which identified both combined and dry-land training as effective strategies for enhancing swimming performance, we conducted a second-tier network meta-analysis to differentiate the relative impact of specific training modalities within these categories. This deeper analysis allowed us to explore which combinations of land- and water-based interventions provide the greatest performance benefits, particularly for competitive swimmers seeking targeted performance gains. The second-tier analysis revealed that W + DRT produced the most favorable outcomes across multiple performance indicators, including 50m and 100 m race times, swimming velocity, stroke rate, and stroke length. Notably, A-HIIT + L-RT consistently ranked second, showing particularly strong effects in sport-specific skills such as stroke rate and swimming velocity. The greater emphasis on high-intensity interval work in water and on land in A-HIIT + DRT likely enhances aerobic capacity and lactate tolerance, making it especially advantageous for middle-distance events (e.g., 200 m), whereas W + DRT’s focus on maximal force production remains optimal for sprint distances (25–100 m). Evidence from aquatic HIIT in clinical and athletic populations (Bunæs-Næss et al., 2023; Tang et al., 2022) and strength-focused RCTs in swimmers (Amara et al., 2021; Amara et al., 2023) supports these differentiated modality effects. These results suggest that while both protocols offer additive benefits over isolated training approaches, W + DRT may provide a more comprehensive stimulus for performance enhancement. Compared to prior literature, our findings extend previous observations by offering the first evidence-based ranking of combined training subtypes. Earlier studies have generally supported the benefits of resistance training in swimmers, with some reporting improvements in muscle power, technical skill, or stroke efficiency (Fone and van den Tillaar, 2022). However, these studies typically evaluated dry-land or aquatic resistance in isolation or relied on simple pre-post comparisons. In contrast, our analysis demonstrates that the concurrent application of resistance in both aquatic and terrestrial environments yields synergistic advantages—a finding that has not been systematically established in previous reviews.

The superiority of W + DRT likely reflects its ability to simultaneously challenge neuromuscular, metabolic, and technical systems in a highly specific and complementary manner. Land-based resistance exercises impose high mechanical loads, promoting muscular strength, power, and intermuscular coordination. In contrast, aquatic resistance provides functional overload within the biomechanical context of swimming, enhancing technical motor patterns under fluid-dynamic conditions. This dual stimulus may be particularly advantageous for sprint events, which demand rapid force production, high intracycle efficiency, and precise movement execution (Guo et al., 2022; Crowley et al., 2017). In comparison, A-HIIT + L-RT—though beneficial—may emphasize cardiovascular conditioning over muscular overload in the aquatic phase, resulting in slightly lower gains in power-related metrics such as stroke length and velocity (Bunæs-Næss et al., 2023). Together, these findings underscore the unique capacity of W + DRT to integrate force development with swimming-specific motor control, making it a superior modality for optimizing multidimensional performance outcomes in swimmers.

Although combined training outperformed single-modality interventions, our first-tier analysis also demonstrated that dry-land training alone produced significant improvements in several performance outcomes, underscoring its continued relevance in swimming-specific conditioning. Given the diversity of dry-land approaches—including strength, power, core, plyometric, and interval training—our second-tier analysis further explored their relative effectiveness to inform evidence-based programming. The results indicated that CT and PT were the most effective dry-land modalities, particularly for enhancing 25 m sprint performance and take-off velocity. These findings support our third major conclusion and suggest that, even within dry-land paradigms, selecting specific training types can yield meaningful performance gains. Previous studies have provided partial support for these findings, with several trials showing positive effects of core or explosive resistance training on swim start dynamics and short-distance race times. Notably, traditional strength (resistance) training—while less dominant in medium-term protocols (4–20 weeks)—may require longer intervention durations to manifest comparable performance improvements, especially in older or more experienced swimmers, where neuromuscular and hypertrophic adaptations accrue over extended periods (Aslam et al., 2025). However, prior meta-analyses often lacked direct comparisons across modalities and did not quantify relative rankings. By employing a network meta-analytic framework, our study offers the first comprehensive comparison, positioning CT and PT above alternatives such as HIIT, PJT, and RT in targeted outcomes (Cui et al., 2025). Mechanistically, core training enhances trunk stability and postural control—key for reducing hydrodynamic drag and maintaining streamlined alignment during starts and high-speed phases (Rodríguez et al., 2025)—whereas power training emphasizes rapid force production crucial for dive starts, breakouts, and turns (van Dijk et al., 2020). Compared to traditional strength or aerobic training, CT and PT may more effectively transfer to swimming-specific neuromuscular demands due to their focus on dynamic stabilization and high-velocity contractions. These adaptations likely explain their superior performance in short-duration, high-intensity events, where hundredths of a second can determine competitive outcomes.

The results of this network meta-analysis provide valuable evidence-based guidance for the design of performance enhancement programs in competitive swimming. Most notably, the consistent superiority of W + DRT underscores the importance of combining high-load dry-land resistance with aquatic-specific resistance exercises to simultaneously target force production, stroke mechanics, and neuromuscular efficiency. This integrated approach should be prioritized in training cycles, particularly for sprinters and youth athletes developing foundational strength and technical capacity. Moreover, the demonstrated benefits of core training and power training offer practical alternatives or adjuncts when water-based resistance tools are unavailable or when individualized programming is needed. These modalities may be especially useful in dry-land blocks, pre-season preparation phases, or in contexts requiring low-equipment solutions. Importantly, the findings support a precision-based and goal-specific approach to physical conditioning in swimming. By identifying the relative effectiveness of various training modalities, coaches and practitioners can move beyond generalized training prescriptions and adopt targeted, evidence-informed strategies tailored to the athlete’s competitive level, stroke specialization, and developmental needs. This aligns with current trends in elite sport toward individualized programming and interdisciplinary collaboration for performance optimization. To enhance transparency and reproducibility, we further translated these results into a practical Standard Operating Procedure (SOP) that specifies decision points, dosage parameters, monitoring indices, and reporting requirements for physical training in swimmers (Table 3).

**TABLE 3:** Standard operating procedure (SOP) for physical training in competitive swimmers.

Component	Recommendations	Evidence basis (NMA)	Notes
Modality	Prioritize W + DRT; include CT/PT for sprints	W + DRT: SUCRA >80% (50 m, 100 m, velocity, stroke metrics); CT/PT: SUCRA 77%–96% (25 m, take-off velocity)	Tailor to event (e.g., sprints)
Frequency	2–4 sessions/week per modality (8–12 weeks)	RCT median: 2-3x/week, 8–12 weeks (e.g., Amara et al., 2022)	Adjust for athlete level
Duration	30–60 min/session	RCT protocols (e.g., 45 min resistance sets)	Include warm-up/cool-down
Intensity	70%–85% 1RM (resistance); RPE 7-9/10 (CT/PT)	Significant MD/SMD (e.g., MD -2.01 s for 100 m, combined training)	Monitor via RPE or velocity
Progression	Increase load/volume 5%–10%weekly; reassess every 4 weeks	RCT progressive overload for sustained gains	Use periodization
Monitoring	Track swim times, velocity, stroke rate/length, start/turn times via biomechanical tools	NMA outcomes (e.g., SMD 1.63 for stroke rate)	Baseline testing; monitor HRV.

Note: NMA, network meta-analysis; SUCRA, surface under the cumulative ranking curve; W + DRT, water plus dand resistance training; CT, core training; PT, power training; RPE, rating of perceived exertion; 1RM, one-repetition maximum; MD, mean difference; SMD, standardized mean difference; RCT, randomized controlled trial; HRV, heart rate variability.

This study possesses several notable strengths. First, it is the first to employ a two-tier network meta-analytic framework to comprehensively compare both general training environments (aquatic, dry-land, combined) and specific physical training modalities, enabling a nuanced, evidence-based hierarchy of interventions for swimming performance. Second, the inclusion of 36 randomized controlled trials involving 844 competitive swimmers enhances the statistical power and external validity of the findings, offering robust guidance applicable across a range of competitive levels and age groups. However, several limitations should be acknowledged. First, despite rigorous methodology, variation in intervention protocols, training duration, and intensity across studies may introduce clinical heterogeneity, potentially influencing effect estimates. Second, the relatively small number of studies for some subgroups—particularly in sport-specific outcomes such as turn time or take-off velocity—may limit the precision of comparisons and the generalizability of subgroup findings. Third, publication bias cannot be entirely excluded, as suggested by visual asymmetry in several funnel plots, although Egger’s tests did not indicate statistically significant bias. Fourth, sex-specific effects could not be assessed because most studies did not provide separate outcome data for male and female swimmers, limiting our ability to determine whether training responses differ by sex. Future high-quality trials with standardized protocols and consistent outcome reporting are warranted to further validate and refine these findings.

## 5 Conclusion

This two-tier network meta-analysis establishes a clear hierarchy of physical training strategies for competitive swimmers. Combined aquatic and dry-land training—particularly W + DRT—demonstrated the greatest overall benefits across both performance metrics and sport-specific skills. Core and power training act as targeted adjuncts to enhance trunk stability and explosive actions in sprint contexts. In practice, a concise guiding principle emerges: pair high-load dry-land work that builds general capacity with in-water, task-specific overload to maximize transfer to performance. Coaches and practitioners are encouraged to prioritize combined resistance modalities and individualize dosage and exercise selection according to athlete profile, event demands, and resource availability.

## Abbreviations

RCT, Randomized Controlled Trial; NMA, Network Meta-Analysis; SUCRA, Surface Under the Cumulative Ranking Curve; MD, Mean Difference; SMD, Standardized Mean Difference; HIIT, High-Intensity Interval Training; PT, Power Training; RT, Resistance Training; CT, Core Training; PJT, Plyometric Jump Training; W + DRT, Water plus Dry-land Resistance Training; A-HIIT + L-RT, Aquatic High-Intensity Interval Training plus Land Resistance Training; RoB 2, Revised Cochrane Risk of Bias Tool; CI, Confidence Interval; CON, Control group.
