# Supplementary material for: Comparative effectiveness of physical training modalities on swimming performance: a two-tier network meta-analysis
Source: Front Physiol. 2025 Aug 28;16:1636595. doi: 10.3389/fphys.2025.1636595 (PMC12423047; doi:10.3389/fphys.2025.1636595)
Supplement: Supplementary file 1 [file Supplementaryfile1.docx]

**Supplementary**

Table of Contents:

**[Supplementary 1: Search Strategy 2](#_Toc159076731)**

[Supplementary 2: Characteristics of studies and subjects included in the review 8](#_Toc159076743)

[Supplementary 3: Risk of Bias 2](#_Toc159076747)7

[Supplementary 4: Publication bias 3](#_Toc159076747)2

# Supplementary 1: Search Strategy

***Search Strategy:***

| #14 | Search: (((((((((swimming[Title/Abstract]) OR (aquatics[Title/Abstract])) OR (Stroke[Title/Abstract])) OR (freestyle[Title/Abstract])) OR (breaststroke[Title/Abstract])) OR (backstroke[Title/Abstract])) OR (butterfly[Title/Abstract])) AND ((((((((((Athletes[MeSH Terms]) OR (Athlete*[Title/Abstract])) OR (Sportsperson[Title/Abstract])) OR (Competitor[Title/Abstract])) OR (Player[Title/Abstract])) OR (Swimmer[Title/Abstract])) OR (Champion[Title/Abstract])) OR (Sportsman[Title/Abstract])) OR (Sportswoman[Title/Abstract])) OR (competitive[Title/Abstract]))) AND (((((((((((((((((((((((((((((((Resistance Training[MeSH Terms]) OR (training[Title/Abstract])) OR ("Strength Training"[Title/Abstract])) OR ("Resistance training"[Title/Abstract])) OR ("Weight training"[Title/Abstract])) OR ("Muscle building"[Title/Abstract])) OR ("Muscle training"[Title/Abstract])) OR ("Power training"[Title/Abstract])) OR ("core training"[Title/Abstract])) OR ("functional training"[Title/Abstract])) OR ("Anaerobic training"[Title/Abstract])) OR ("Resistance exercise"[Title/Abstract])) OR ("Strength conditioning"[Title/Abstract])) OR ("Neuromuscular training"[Title/Abstract])) OR ("muscular training"[Title/Abstract])) OR (Training, Resistance[Title/Abstract])) OR (Training, Strength[Title/Abstract])) OR (Weight-Lifting Strengthening Program[Title/Abstract])) OR (Strengthening Programs, Weight-Lifting[Title/Abstract])) OR (Strengthening Program, Weight-Lifting[Title/Abstract])) OR (Weight Lifting Strengthening Program[Title/Abstract])) OR (Weight-Lifting Strengthening Programs[Title/Abstract])) OR (Weight-Lifting Exercise Program[Title/Abstract])) OR (Exercise Programs, Weight-Lifting[Title/Abstract])) OR (Exercise Program, Weight-Lifting[Title/Abstract])) OR (Weight Lifting Exercise Program[Title/Abstract])) OR (Weight-Lifting Exercise Programs[Title/Abstract])) OR (Weight-Bearing Strengthening Program[Title/Abstract])) OR (Strengthening Programs, Weight-Bearin[Title/Abstract])) OR (Weight Bearing Exercise Program[Title/Abstract])) OR (((((((((((((((((((((((((((Plyometric exercise[MeSH Terms]) OR (Exercise, Plyometric[Title/Abstract])) OR (Exercises, Plyometric[Title/Abstract])) OR (Plyometric Exercises[Title/Abstract])) OR (Plyometric Training[Title/Abstract])) OR (Plyometric Trainings[Title/Abstract])) OR (Training, Plyometric[Title/Abstract])) OR (Trainings, Plyometric[Title/Abstract])) OR (Plyometric Drill[Title/Abstract])) OR (Plyometric Drills[Title/Abstract])) OR (Stretch-Shortening Exercise[Title/Abstract])) OR (Exercises, Stretch-Shortening[Title/Abstract])) OR (Exercise, Stretch-Shortening[Title/Abstract])) OR (Stretch Shortening Exercise[Title/Abstract])) OR (Stretch-Shortening Exercises[Title/Abstract])) OR (Stretch-Shortening Drill[Title/Abstract])) OR (Drills, Stretch-Shortening[Title/Abstract])) OR (Drill, Stretch-Shortening[Title/Abstract])) OR (Stretch Shortening Drill[Title/Abstract])) OR (Stretch-Shortening Drills[Title/Abstract])) OR (Stretch-Shortening Cycle Exercise[Title/Abstract])) OR (Cycle Exercises, Stretch-Shortening[Title/Abstract])) OR (Cycle Exercise, Stretch-Shortening[Title/Abstract])) OR (Exercises, Stretch-Shortening Cycle[Title/Abstract])) OR (Exercise, Stretch-Shortening Cycle[Title/Abstract])) OR (Stretch Shortening Cycle Exercise[Title/Abstract])) OR (Stretch-Shortening Cycle Exercises[Title/Abstract])))) AND ((randomized controlled trial[pt] OR controlled clinical trial[pt] OR randomized[tiab] OR placebo[tiab] OR drug therapy[sh] OR randomly[tiab] OR trial[tiab] OR groups[tiab]) NOT (animals[mh] NOT humans[mh])) |
| --- | --- |
| #13 | Search: (randomized controlled trial[pt] OR controlled clinical trial[pt] OR randomized[tiab] OR placebo[tiab] OR drug therapy[sh] OR randomly[tiab] OR trial[tiab] OR groups[tiab]) NOT (animals[mh] NOT humans[mh]) |
| #12 | Search: ((((((((((((((((((((((((((((((Resistance Training[MeSH Terms]) OR (training[Title/Abstract])) OR ("Strength Training"[Title/Abstract])) OR ("Resistance training"[Title/Abstract])) OR ("Weight training"[Title/Abstract])) OR ("Muscle building"[Title/Abstract])) OR ("Muscle training"[Title/Abstract])) OR ("Power training"[Title/Abstract])) OR ("core training"[Title/Abstract])) OR ("functional training"[Title/Abstract])) OR ("Anaerobic training"[Title/Abstract])) OR ("Resistance exercise"[Title/Abstract])) OR ("Strength conditioning"[Title/Abstract])) OR ("Neuromuscular training"[Title/Abstract])) OR ("muscular training"[Title/Abstract])) OR (Training, Resistance[Title/Abstract])) OR (Training, Strength[Title/Abstract])) OR (Weight-Lifting Strengthening Program[Title/Abstract])) OR (Strengthening Programs, Weight-Lifting[Title/Abstract])) OR (Strengthening Program, Weight-Lifting[Title/Abstract])) OR (Weight Lifting Strengthening Program[Title/Abstract])) OR (Weight-Lifting Strengthening Programs[Title/Abstract])) OR (Weight-Lifting Exercise Program[Title/Abstract])) OR (Exercise Programs, Weight-Lifting[Title/Abstract])) OR (Exercise Program, Weight-Lifting[Title/Abstract])) OR (Weight Lifting Exercise Program[Title/Abstract])) OR (Weight-Lifting Exercise Programs[Title/Abstract])) OR (Weight-Bearing Strengthening Program[Title/Abstract])) OR (Strengthening Programs, Weight-Bearin[Title/Abstract])) OR (Weight Bearing Exercise Program[Title/Abstract])) OR (((((((((((((((((((((((((((Plyometric exercise[MeSH Terms]) OR (Exercise, Plyometric[Title/Abstract])) OR (Exercises, Plyometric[Title/Abstract])) OR (Plyometric Exercises[Title/Abstract])) OR (Plyometric Training[Title/Abstract])) OR (Plyometric Trainings[Title/Abstract])) OR (Training, Plyometric[Title/Abstract])) OR (Trainings, Plyometric[Title/Abstract])) OR (Plyometric Drill[Title/Abstract])) OR (Plyometric Drills[Title/Abstract])) OR (Stretch-Shortening Exercise[Title/Abstract])) OR (Exercises, Stretch-Shortening[Title/Abstract])) OR (Exercise, Stretch-Shortening[Title/Abstract])) OR (Stretch Shortening Exercise[Title/Abstract])) OR (Stretch-Shortening Exercises[Title/Abstract])) OR (Stretch-Shortening Drill[Title/Abstract])) OR (Drills, Stretch-Shortening[Title/Abstract])) OR (Drill, Stretch-Shortening[Title/Abstract])) OR (Stretch Shortening Drill[Title/Abstract])) OR (Stretch-Shortening Drills[Title/Abstract])) OR (Stretch-Shortening Cycle Exercise[Title/Abstract])) OR (Cycle Exercises, Stretch-Shortening[Title/Abstract])) OR (Cycle Exercise, Stretch-Shortening[Title/Abstract])) OR (Exercises, Stretch-Shortening Cycle[Title/Abstract])) OR (Exercise, Stretch-Shortening Cycle[Title/Abstract])) OR (Stretch Shortening Cycle Exercise[Title/Abstract])) OR (Stretch-Shortening Cycle Exercises[Title/Abstract])) |
| #11 | Search: ((((((((((((((((((((((((((Plyometric exercise[MeSH Terms]) OR (Exercise, Plyometric[Title/Abstract])) OR (Exercises, Plyometric[Title/Abstract])) OR (Plyometric Exercises[Title/Abstract])) OR (Plyometric Training[Title/Abstract])) OR (Plyometric Trainings[Title/Abstract])) OR (Training, Plyometric[Title/Abstract])) OR (Trainings, Plyometric[Title/Abstract])) OR (Plyometric Drill[Title/Abstract])) OR (Plyometric Drills[Title/Abstract])) OR (Stretch-Shortening Exercise[Title/Abstract])) OR (Exercises, Stretch-Shortening[Title/Abstract])) OR (Exercise, Stretch-Shortening[Title/Abstract])) OR (Stretch Shortening Exercise[Title/Abstract])) OR (Stretch-Shortening Exercises[Title/Abstract])) OR (Stretch-Shortening Drill[Title/Abstract])) OR (Drills, Stretch-Shortening[Title/Abstract])) OR (Drill, Stretch-Shortening[Title/Abstract])) OR (Stretch Shortening Drill[Title/Abstract])) OR (Stretch-Shortening Drills[Title/Abstract])) OR (Stretch-Shortening Cycle Exercise[Title/Abstract])) OR (Cycle Exercises, Stretch-Shortening[Title/Abstract])) OR (Cycle Exercise, Stretch-Shortening[Title/Abstract])) OR (Exercises, Stretch-Shortening Cycle[Title/Abstract])) OR (Exercise, Stretch-Shortening Cycle[Title/Abstract])) OR (Stretch Shortening Cycle Exercise[Title/Abstract])) OR (Stretch-Shortening Cycle Exercises[Title/Abstract]) |
| #10 | Search: (Exercise, Plyometric[Title/Abstract])) OR (Exercises, Plyometric[Title/Abstract])) OR (Plyometric Exercises[Title/Abstract])) OR (Plyometric Training[Title/Abstract])) OR (Plyometric Trainings[Title/Abstract])) OR (Training, Plyometric[Title/Abstract])) OR (Trainings, Plyometric[Title/Abstract])) OR (Plyometric Drill[Title/Abstract])) OR (Plyometric Drills[Title/Abstract])) OR (Stretch-Shortening Exercise[Title/Abstract])) OR (Exercises, Stretch-Shortening[Title/Abstract])) OR (Exercise, Stretch-Shortening[Title/Abstract])) OR (Stretch Shortening Exercise[Title/Abstract])) OR (Stretch-Shortening Exercises[Title/Abstract])) OR (Stretch-Shortening Drill[Title/Abstract])) OR (Drills, Stretch-Shortening[Title/Abstract])) OR (Drill, Stretch-Shortening[Title/Abstract])) OR (Stretch Shortening Drill[Title/Abstract])) OR (Stretch-Shortening Drills[Title/Abstract])) OR (Stretch-Shortening Cycle Exercise[Title/Abstract])) OR (Cycle Exercises, Stretch-Shortening[Title/Abstract])) OR (Cycle Exercise, Stretch-Shortening[Title/Abstract])) OR (Exercises, Stretch-Shortening Cycle[Title/Abstract])) OR (Exercise, Stretch-Shortening Cycle[Title/Abstract])) OR (Stretch Shortening Cycle Exercise[Title/Abstract])) OR (Stretch-Shortening Cycle Exercises[Title/Abstract]) |
| #9 | Search: Plyometric exercise[MeSH Terms] |
| #8 | Search: (((((((((((((((((((((((((((((Resistance Training[MeSH Terms]) OR (training[Title/Abstract])) OR ("Strength Training"[Title/Abstract])) OR ("Resistance training"[Title/Abstract])) OR ("Weight training"[Title/Abstract])) OR ("Muscle building"[Title/Abstract])) OR ("Muscle training"[Title/Abstract])) OR ("Power training"[Title/Abstract])) OR ("core training"[Title/Abstract])) OR ("functional training"[Title/Abstract])) OR ("Anaerobic training"[Title/Abstract])) OR ("Resistance exercise"[Title/Abstract])) OR ("Strength conditioning"[Title/Abstract])) OR ("Neuromuscular training"[Title/Abstract])) OR ("muscular training"[Title/Abstract])) OR (Training, Resistance[Title/Abstract])) OR (Training, Strength[Title/Abstract])) OR (Weight-Lifting Strengthening Program[Title/Abstract])) OR (Strengthening Programs, Weight-Lifting[Title/Abstract])) OR (Strengthening Program, Weight-Lifting[Title/Abstract])) OR (Weight Lifting Strengthening Program[Title/Abstract])) OR (Weight-Lifting Strengthening Programs[Title/Abstract])) OR (Weight-Lifting Exercise Program[Title/Abstract])) OR (Exercise Programs, Weight-Lifting[Title/Abstract])) OR (Exercise Program, Weight-Lifting[Title/Abstract])) OR (Weight Lifting Exercise Program[Title/Abstract])) OR (Weight-Lifting Exercise Programs[Title/Abstract])) OR (Weight-Bearing Strengthening Program[Title/Abstract])) OR (Strengthening Programs, Weight-Bearin[Title/Abstract])) OR (Weight Bearing Exercise Program[Title/Abstract]) |
| #7 | Search: (training[Title/Abstract])) OR ("Strength Training"[Title/Abstract])) OR ("Resistance training"[Title/Abstract])) OR ("Weight training"[Title/Abstract])) OR ("Muscle building"[Title/Abstract])) OR ("Muscle training"[Title/Abstract])) OR ("Power training"[Title/Abstract])) OR ("core training"[Title/Abstract])) OR ("functional training"[Title/Abstract])) OR ("Anaerobic training"[Title/Abstract])) OR ("Resistance exercise"[Title/Abstract])) OR ("Strength conditioning"[Title/Abstract])) OR ("Neuromuscular training"[Title/Abstract])) OR ("muscular training"[Title/Abstract])) OR (Training, Resistance[Title/Abstract])) OR (Training, Strength[Title/Abstract])) OR (Weight-Lifting Strengthening Program[Title/Abstract])) OR (Strengthening Programs, Weight-Lifting[Title/Abstract])) OR (Strengthening Program, Weight-Lifting[Title/Abstract])) OR (Weight Lifting Strengthening Program[Title/Abstract])) OR (Weight-Lifting Strengthening Programs[Title/Abstract])) OR (Weight-Lifting Exercise Program[Title/Abstract])) OR (Exercise Programs, Weight-Lifting[Title/Abstract])) OR (Exercise Program, Weight-Lifting[Title/Abstract])) OR (Weight Lifting Exercise Program[Title/Abstract])) OR (Weight-Lifting Exercise Programs[Title/Abstract])) OR (Weight-Bearing Strengthening Program[Title/Abstract])) OR (Strengthening Programs, Weight-Bearin[Title/Abstract])) OR (Weight Bearing Exercise Program[Title/Abstract]) |
| #6 | Search: Resistance Training[MeSH Terms] |
| #5 | Search: (((((((swimming[Title/Abstract]) OR (aquatics[Title/Abstract])) OR (Stroke[Title/Abstract])) OR (freestyle[Title/Abstract])) OR (breaststroke[Title/Abstract])) OR (backstroke[Title/Abstract])) OR (butterfly[Title/Abstract])) AND ((((((((((Athletes[MeSH Terms]) OR (Athlete*[Title/Abstract])) OR (Sportsperson[Title/Abstract])) OR (Competitor[Title/Abstract])) OR (Player[Title/Abstract])) OR (Swimmer[Title/Abstract])) OR (Champion[Title/Abstract])) OR (Sportsman[Title/Abstract])) OR (Sportswoman[Title/Abstract])) OR (competitive[Title/Abstract])) |
| #4 | Search: (((((((((Athletes[MeSH Terms]) OR (Athlete*[Title/Abstract])) OR (Sportsperson[Title/Abstract])) OR (Competitor[Title/Abstract])) OR (Player[Title/Abstract])) OR (Swimmer[Title/Abstract])) OR (Champion[Title/Abstract])) OR (Sportsman[Title/Abstract])) OR (Sportswoman[Title/Abstract])) OR (competitive[Title/Abstract]) |
| #3 | Search: (Athlete*[Title/Abstract])) OR (Sportsperson[Title/Abstract])) OR (Competitor[Title/Abstract])) OR (Player[Title/Abstract])) OR (Swimmer[Title/Abstract])) OR (Champion[Title/Abstract])) OR (Sportsman[Title/Abstract])) OR (Sportswoman[Title/Abstract])) OR (competitive[Title/Abstract]) |
| #2 | Search: Athletes[MeSH Terms] |
| #1 | Search: ((((((swimming[Title/Abstract]) OR (aquatics[Title/Abstract])) OR (Stroke[Title/Abstract])) OR (freestyle[Title/Abstract])) OR (breaststroke[Title/Abstract])) OR (backstroke[Title/Abstract])) OR (butterfly[Title/Abstract]) |

# Supplementary 2: Characteristics of studies and subjects included in the review

## Table 2.1: Characteristics of subjects included in the review

| **Study** | **Country/Region** | **Athlete Level** | **Subjects  (intervention/ control)** | **Sex (male/female) (intervention/ control)** | **Mean age  (intervention/ control)** | **Height (cm)** | **Body mass (kg)** | **Swimming experience (years)** |
| --- | --- | --- | --- | --- | --- | --- | --- | --- |
|  |  |  |  |  |  |  |  |  |
| Amara et al. (2021b) | Tunisia | Competitive swimmers | 22 (11/11) | 11/0 vs. 11/0 | 16.5 ± 0.30 vs. 16.1 ± 0.32 | 174 ± 9.80 vs. 175 ± 9.70 | 72.7 ± 5.30 vs. 73.6 ± 5.25 | 6.86 ± 0.33 vs. 6.78 ± 0.34 |
| Amara et al. (2022) | Tunisia | Competitive swimmers | 22 (11/11) | 11/0 vs. 11/0 | 16.5 ± 0.3 vs. 16.1 ± 0.3 | 174 ± 9.80 vs. 175 ± 9.70 | 72.7 ± 5.30 vs. 73.6 ± 5.25 | NA |
| Born et al. (2020) | Switzerland, Austria, Hungary | Young competitive swimmer | 21 (10/11) | 6/4 vs. 3/8 | 17.1 ± 2.6 vs. 17.1 ± 2.7 | 175 ± 10 vs. 172 ± 7 | 65.8 ± 10.1 vs. 62.9 ± 9.1 | NA |
| Amara et al. (2023) | Tunisia | Competitive swimmers | 22 (11/11) | 11/0 vs. 11/0 | 14.1 ± 0.30 vs. 14.5 ± 0.32 | 170 ± 9.8 vs. 171 ± 8.4 | 68.74 ± 4.35 vs. 68.08 ± 3.79 | 6.43 ± 0.29 vs. 6.34 ± 0.25 |
| Amaro et al. (2017) | Portugal | Competitive swimmers | 21 (7/7/7) | 7/0 vs. 7/0 vs. 7/0 | 12.7 ± 0.8 vs. 12.7 ± 0.8 vs. 12.6 ± 0.8 | 157 ± 7 vs. 158 ± 9 vs. 155 ± 7 | 47.9 ± 7.2 vs. 47.4 ± 10.0 vs. 47.8 ± 12.8 | ≥2 years |
| Aouani et al. (2024) | Tunisia | National-level competitive swimmers | 22 (11/11) | 11/0 vs. 11/0 | 16.5 ± 0.29 vs. 16.1 ± 0.33 | 177 ± 8.82 vs. 176 ± 8.40 | 74.5 ± 5.36 vs. 74.6 ± 5.11 | >6 years |
| Aspenes et al. (2009) | Norway | Competitive swimmers | 20 (11/9) | 5/6 vs. 2/7 | 17.5 ± 2.9 vs. 15.9 ± 1.1 | 171 ± 9 vs. 173 ± 6 | 58.9 ± 10.2 vs. 58.3 ± 6.6 | NA |
| Bishop et al. (2009) | UK | Young competitive swimmer | 22 (11/11) | NA | 13.1 ± 1.4 vs. 12.6 ± 1.9 | 162.9 ± 11.9 vs. 157.6 ± 11.9 | 50.6 ± 12.3 vs. 43.3 ± 11.6 | 3.4 ± 1.8 vs. 3.8 ± 2.3 |
| Breed et al. (2003) | Australia | Young competitive swimmer | 23 (12/11) | 0/12 vs. 0/11 | 18.9 ± 1.5 | 166 ± 7 | 64.9 ± 5.2 | NA |
| Cañas-Jamett et al. (2020) | Chile | Competitive amateur swimmers | 18 (8/10) | 8/0 vs. 10/0 | 20.5 ± 0.6 vs. 20.1 ± 0.7 | 176 ± 4 vs. 178 ± 5 | 74.8 ± 4.9 vs. 75.4 ± 5.4 | 2.8 ± 0.75 vs. 2.7 ± 0.9 |
| Chortane et al. (2022) | Tunisia | Competitive swimmers | 28 (14/14) | 14/0 vs. 14/0 | 16.4 ± 0.31 vs. 16.1 ± 0.30 | 173 ± 9.82 vs. 175 ± 9.40 | 72.5 ± 5.36 vs. 74.6 ± 5.11 | >3 years |
| Cossor et al. (1999) | Australia | Young competitive swimmer | 38 (19/19) | NA | 11.7 ± 1.16 | 159.1 ± 7.8 vs. 154.7 ± 8.4 | 47.4 ± 10.8 vs. 44.0 ± 7.1 | NA |
| Czuba et al. (2017) | Poland | National-level competitive swimmers | 16 (8/8) | 8/0 vs. 8/0 | 21.4 ± 2.1 vs. 21.1 ± 1.9 | 183.3 ± 5.2 | 75.9 ± 6.1 | >6 years |
| Dragunas et al. (2012) | Canada | Competitive collegiate swimmers | 18 (9/9) | 5/4 vs. 5/4 | 19.3 ± 0.87 vs. 19.0 ± 1.80 | NA | NA | NA |
| Garrido et al. (2010) | Portugal | Competitive swimmers | 23 (12/11) | 8/4 vs. 6/5 | 12.0±0.78 vs. 12.18±0.75 | 151 ± 0.04 vs. 152 ± 0.06 | 41.29 ± 8.05 vs. 43.40 ± 7.66 | NA |
| Gencer et al. (2018) | Turkey | Competitive amateur swimmers | 24 (12/12) | 0/12 vs. 0/12 | 10.58±1.31 vs. 10.75±1.29 | 140.92±13.76 vs. 145.08±12.17 | 36.37±7.65 vs. 38.58±5.17 | 3 years |
| Girold et al. (2006) | France | Competitive swimmers | 37 (15/11/11) | 16/21 | 16.5±2 vs. 18±3 vs. 17±3 | 170±7 vs. 176±7 vs. 168±9 | 58±9.5 vs. 67±10.5 vs. 62±11 | NA |
| Girold et al. (2007) | France | Competitive swimmers | 21 (7/7/7) | 10/11 | 16.5 ± 2.5 vs. 16.5 ± 2.5 vs. 16.5 ± 1.5 | 171 ± 9 vs. 170 ± 11 vs. 171 ± 11 | 64 ± 8 vs. 62 ± 4 vs. 62 ± 4 | 5-6 years |
| Girold et al. (2012) | France | Competitive swimmers | 24 (8/8/8) | 12/12 | 21.1±1.4 vs. 19.5±1.5 vs.24.2±4.6 | 176±8 vs. 170±11 vs. 175±7 | 69.1±6.5 vs. 62.1±9.8 vs. 69.3±7.4 | >4 years |
| Gourgoulis et al. (2019) | Greece | Competitive swimmers | 12 (6/6) | 0/6 vs. 0/6 | 13.08±0.9 | 158 ± 5 cm | 48.3 ± 6.9 kg | 3.92 ± 0.9 |
| Karpiński et al. (2020) | Poland | Competitive swimmers | 16 (8/8) | 8/0 vs. 8/0 | 20.2 ± 1.17 vs. 20.0 ± 1.9 | 183.0 ± 6.57 vs. 182.1 ± 3.18 | 74.9 ± 10.67 vs. 75.4 ± 6.27 | ≥10 years |
| Khiyami et al. (2022) | Saudi Arabia | Young competitive swimmer | 18 (9/9) | 9/0 vs. 9/0 | 13 ± 2 vs. 13.11 ± 2.6 | 158.8 ± 17.3 vs. 160.4 ± 11.9 | 48.3 ± 14.2 vs. 49.1 ± 11.3 | 2.8 ± 0.4 vs. 2.9 ± 0.7 |
| Kilen et al. (2014) | Denmark | National-level competitive swimmers | 41 (20/21) | 14/6 vs. 16/5 | 20.0±2.7 vs. 20.0±2.7 | 179.9 ± 6.5 | 72.0 ± 10.6 | ≥5 years |
| Lopes et al. (2021) | Portugal | Competitive collegiate swimmers | 20 (11/9) | 9/2 vs. 5/4 | 20.45±1.63 vs. 20.67±2.00 | 79 ± 5 vs. 174.33 ± 8 | 70.76 ± 6.15 vs. 66.52 ± 9.06 | NA |
| Naczk et al. (2016) | Poland | National-level competitive swimmers | 14 (7/7) | 5/2 vs. 5/2 | 15.8±0.4 | 179±7 | 69±8 | ≥6 years |
| Norberto et al. (2023) | Brazil | Young competitive swimmer | 29 (11/7/7) | 7/4 vs. 4/3 vs. 5/2 | 15.6±2.1 | 164.7±5.6 | 58.8±4.4 | ≥2 years |
| Nugent et al. (2018) | Ireland | Young competitive swimmer | 15 (7/8) | 3/4 vs. 3/5 | 16.0±1.1 vs. 15.6±0.9 | 174.9±9.3 vs. 172.3±10.0 | 66.3±10.6 vs. 65.3±12.5 | NA |
| Patil et al. (2014) | India | Young competitive swimmer | 60 (30/30) | 19/11 vs. 19/11 | 14.7±1.29 vs. 13.4±1.50 | NA | NA | ≥2 years |
| Potdevin et al. (2011) | France | Young competitive swimmer | 23 (12/11) | 7/5 vs. 6/5 | 14.3 ± 0.2 vs. 14.1 ± 0.2 | 161±12 vs. 158±12 | 50.03±9.04 vs. 50.85±12.71 | ≥3 years |
| Sadowski et al. (2020) | Poland | Young competitive swimmer | 26 (12/14) | 12/0 vs. 14/0 | 15.8 ± 0.4 vs. 15.6 ± 0.6 | 175.7 ± 5.9 vs. 173.4 ± 7.1 | 67.8 ± 7.9 vs. 69.1 ± 8.4 | 5.8 ± 0.7 |
| Sammoud et al. (2019) | Tunisia | Young competitive swimmer | 26 (14/12) | 14/0 vs. 12/0 | 10.3±0.4 vs. 10.5±0.4 | 143±8 vs. 146±7 | 36.2±8.4 vs. 38.2±5.9 | 2.0±1.6 |
| Sammoud et al. (2021) | Tunisia | Young competitive swimmer | 22 (12/10) | 12/0 vs. 10/0 | 10.01 ± 0.57 vs. 10.50 ± 0.28 | 146.90 ± 7.62 vs. 143.60 ± 5.05 | 36.39 ± 6.32 vs. 38.41 ± 9.42 | 2.0 ± 1.4 |
| Sperlich et al. (2010) | Germany, Norway, Sweden | Young competitive swimmer | 26 (13/13) | 13/0 vs. 13/0 | 10.5 ± 1.4 vs. 10.5 ± 1.4 | 156.5 ± 8.1 | 45.2 ± 9.7 | ≥3 years |
| Toussaint et al. (1990) | Netherlands | Competitive swimmers | 22 (11/11) | 8/3 vs. 8/3 | 18.40 ± 2.10 vs. 18.50 ± 3.30 | 179 ± 7 vs. 178 ± 8 | 72.3 ± 8.0 vs. 69.2 ± 7.8 | ≥5 years |
| Weston et al. (2015) | UK | Young competitive swimmer | 20 (10/10) | 5/5 vs. 5/5 | 15.7 ± 1.2 vs. 16.7 ± 0.9 | 172 ± 6 vs. 170 ± 3 | 63 ± 5 vs. 63 ± 3 | ≥3 years |
| Jones et al. (2017) | Australia | National-level competitive swimmers | 12 (6/6) | 4/2 vs. 6/0 | 19.4 ± 1.1 vs. 18.9 ± 0.9 | 179.1 ± 8.6 vs. 178.0 ± 10.4 | 78.9 ± 12.3 vs. 77.1 ± 10.2 | 8.3 ± 3.1 vs. 8.9 ± 3.5 |

Note: N/A, Not Applicable.

## Table 2.2 Characteristics of interventions and outcomes included in the review

| **Study** | **Intervention detail** | | **Intensity** | **Time (minutes)** | **Frequency** | **Duration (weeks)** | **Outcomes** |
| --- | --- | --- | --- | --- | --- | --- | --- |
|  | **Intervention group** | **Control group** |  |  |  |  |  |
| Amara et al. (2021b) | Water resistance training (hand paddles + water resistance parachute) + land resistance training (bench press + medicine ball throwing) | Conventional water training (low/high intensity aerobic + HIIT) | Dry land training: 60%-80% 1RM BP (bench press), medicine ball weight 2-5 kg. Water resistance training: resistance series training at maximum speed. | Dry training: 60-75 minutes/time. Water training: 90-120 minutes/time. | Land training 2 times a week, water training 6 times a week | 6 weeks | 25 m performance, 50 m performance, Swimming velocity, Stroke rate, Stroke length |
| Amara et al. (2022) | Water resistance (using a small drag parachute) + land resistance (Back squat, CMJ, CMJ box) | General training, including general strength training and water training | Back squat: 60–80% 1RM; Water resistance swimming: all-out sprint with 288 cm² water umbrella | Dry training: 90–120 min Water training: 60–75 min | Land training 2 times a week, water training 6 times a week Control 2 times a week | 9 weeks | 100 m performance, Turn time, Start time |
| Born et al. (2020) | Heavy-load back squat and deadlift exercises were performed twice weekly, with 3–4 sets of 2–8 repetitions per session and progressively increasing loads. | Unloaded box jumps, including countermovement and squat jumps, were conducted twice weekly, with sets and repetitions adjusted according to training progression. | (a) The squat increased from 43±11 kg to 65±12 kg, and the deadlift increased from 48±15 kg to 79±20 kg. (b) The box height increased from 75±9 cm to 96±7 cm. | 60 min | 2 times/week | 6 weeks | Take-off velocity |
| Amara et al. (2023) | HIIT (pool) + maximum strength training (bench press, leg extension) | Conventional training (low intensity high volume + core training) | EG：85–95% 1RM； CG：70–85% HRmax | 60-75 min | Land training 2 times a week, water training 6 times a week | 8 weeks | 100 m performance, Swimming velocity, Stroke rate, Stroke length |
| Amaro et al. (2017) | (a) Regular strength training, including exercises such as medicine ball throws, reverse jumps, dumbbell flyes, Russian twists, push-ups, etc. (b) Same exercises as GR1, focusing on explosive power, requiring you to complete as many repetitions as possible within the allotted time. | Swimming training only | NA | 30 min | 2 times/week | 6 weeks | 50 m performance |
| Aouani et al. (2024) | HIT group: 40% high intensity + 60% aerobic | Conventional training group: 90% aerobic + 10% HIT | 80–95% HRmax | 80 min | 6 times/week | 2 weeks | 100 m performance |
| Aspenes et al. (2009) | High-intensity interval training (4×4min, 90–95% HRmax) + maximum strength training (shoulder pulldown) | Regular training | HIIT: 90–95% HRmax Maximum strength training: 5RM | 30 min | HIIT: 2 times/week Maximum strength training: 2 times/week | 11 weeks | 50 m performance, 100 m performance |
| Bishop et al. (2009) | Twice-weekly land-based plyometric training, including jump-based and reactive power exercises. | Maintained habitual swim training without any additional land-based exercises. | Progressively intensified with low-, moderate-, and high-impact plyometric drills introduced weekly. | 60 min | 2 times/week | 8 weeks | Take-off velocity, Start time |
| Breed et al. (2003) | 9-week resistance training program, 3 times per week, with the main goal of improving vertical jump ability (clean squats, jump squats, shoulder presses, weighted jumps, weighted squats, etc.) | Regular training | RM setting increment | 45-60 min | 3 times/week | 9 weeks | Take-off velocity |
| Cañas-Jamett et al. (2020) | High-intensity plyometric training on land for 6 weeks, ~15 minutes each time, replacing the end of the original swimming training | Regular speed swimming training | Progressively increased box height (20–60 cm) | 15 min | 2 times/week | 6 weeks | 200 m performance |
| Chortane et al. (2022) | High-volume aquatic training: 132.4 km；75% aerobic training, 15% HIIT, 10% HIT | Standard moderate-volume training | Aerobic: 50%–75% HRmax HIIT: 75%–85% HRmax HIT: 85%–95% HRmax | 90–122 min | NA | 4 weeks | 50 m performance |
| Cossor et al. (1999) | Plyometric exercises, 30 minutes per day, 3 times per week, including 15 exercises, 2 sets of 10-15 reps per set. | Regular training | Low to moderate intensity | 15 min | 3 times/week | 20 weeks | 50 m performance, Take-off velocity, Turn time, Start time |
| Czuba et al. (2017) | High-intensity interval training (HIIT): HIIT was performed using a power bicycle, with a training frequency of 2 times per week for 8 weeks; the intervention duration was 30 minutes/time. | Regular swimming training (9 times per week, total training volume unchanged) | 95% VO₂max | 30 min | 2 times/week | 8 weeks | 50 m performance, 100 m performance, 200 m performance |
| Dragunas et al. (2012) | Training was performed wearing a resistance suit (TYR 2.0 drag suit) in a specific training group (3 training units) 3 times a week for 5 weeks. The training content included short-distance high-intensity sprints, such as 3 rounds of 30m+20m+10m sprints, 8×25m sprints, 3×100m sprints, etc. | The subjects performed the same training content and intensity as the intervention group, but wore regular training clothes. | Short distance high intensity | 30-45 min | 3 times/week | 5 weeks | 50 m performance, Stroke rate, Stroke length |
| Garrido et al. (2010) | Dry-land strength training was performed twice a week for 8 weeks. Strength training included bench press, leg extension, countermovement jump (CMJ), and medicine ball throwing (1 kg). | Regular swimming training | 50–75% 6-RM | 20 min | 2 times/week | 8+6 weeks | 25 m performance, 50 m performance |
| Gencer et al. (2018) | Core training: 8 weeks of core training (20 minutes each, low intensity, fixed movements, repetitive), 5 times a week, before regular swimming training | Regular swimming training | Low-intensity | 20 min | 5 times/week | 8 weeks | 25 m performance, 50 m performance |
| Girold et al. (2006) | (a) Resisted Sprint Training: Using elastic bands to provide reverse resistance (pulling the athlete from the starting block), the athlete must overcome the resistance to complete the stroke; (b) Assisted Sprint Training: Using elastic bands to pull athletes forward, they are accelerated while swimming | Regular swimming training | maximal speed | 6 min | 3 times/week | 3 weeks | 100 m performance, Stroke rate, Stroke length |
| Girold et al. (2007) | (a) Dry land strength training is done twice a week, 45 minutes each time, focusing on training the upper limbs, abdomen and lower limb muscles, using barbells for exercises such as bench presses, pull-ups and squats. (b) There are resistance and assisted sprint training in the water. RAS training is 2 times a week, 45 minutes each time, using elastic tubing for resistance and assisted sprint exercises | Regular training | (a) 80-90% 1RM (b) maximal speed | 45 min | 6 times/week | 12 weeks | 50 m performance, Stroke rate, Stroke length |
| Girold et al. (2012) | (a) Land-based strength training 3 times per week (upper body resistance training, including pull-ups, cable training, intensity 80–90% 1RM) (b) Electrical stimulation training 3 times per week, using an electrical stimulator (stimulating the latissimus dorsi) at an intensity controlled at 80–90% of perceived maximum voluntary contraction | Regular swimming training | 80-90% 1RM | 15 min | 3 times/week | 4 weeks | 50 m performance, Stroke rate, Stroke length |
| Gourgoulis et al. (2019) | Water resistance training, using a water parachute to add resistance. | Regular swimming training | Maximal intensity sprints | 15-30 min | 4 times/week | 11 weeks | 50 m performance, 100 m performance, 200 m performance, Swimming velocity, Stroke rate, Stroke length |
| Karpiński et al. (2020) | 6 weeks, 3 times per week land-based core muscle group training (SCMT), added to the regular training, including flutter kicks, single leg V-ups, prone physio ball trunk extension and Russian twists | Regular training (10 water training sessions + 2 strength training sessions per week) | Increase the number of training weeks, including weight, position changes, unstable surfaces (such as wiggle cushion), etc. | 25 min | 3 times/week | 6 weeks | 50 m performance, Take-off velocity, Turn time, Start time, Swimming velocity, Stroke rate, Stroke length |
| Khiyami et al. (2022) | The pre-training group added core training to the regular training for 6 weeks, 3 times a week, about 1 hour each time, including 7 core training movements (such as plank support, bird dog, leg press, side bridge, etc.) | Regular swimming training | Mainly bodyweight training, some projects use 3-7 kg weight progression | 60 min | 3 times/week | 6 weeks | 50 m performance, Swimming velocity, Stroke rate, Stroke length |
| Kilen et al. (2014) | High-intensity interval training (HIT), which consists of 6-10 10-30 second all-out sprints, followed by 2-4 minute rests | Regular training plan (~35km/week, ~12 hours/week) | maximal speed | 30-45 min | 6-8 times/week | 12 weeks | 100 m performance, 200 m performance |
| Lopes et al. (2021) | 1 land strength training per week (bench press, full squat, CMJ, CMJ with free arms, medicine ball throw) + regular swimming training | Regular swimming training | 80-90% 1RM | 45-60 min | 1 time/week | 8 weeks | 50 m performance, 100 m performance |
| Naczk et al. (2016) | Inertial Training (IT), using the Inertial Training Measurement System (ITMS), targets muscles involved in the upward sweep phase of the freestyle and butterfly strokes. | Regular swimming training | maximal effort | 5-10 min (60 seconds (4 sets x 15 seconds)) | 3 times/week | 4 weeks | 50 m performance, 100 m performance |
| Norberto et al. (2023) | (a) Combine strength and power training, including free weight training (barbell squat clean and press, plyometric bench-press, etc.) (b) Strength training only, using machines (such as machine bench-press, lat pulldown, etc.) | Regular swimming training | 30–95% 1RM | 50 min | 3 times/week | 6 weeks | 50 m performance, 100 m performance, 200 m performance |
| Nugent et al. (2018) | HIT: 6 hours of training per week (17.0 km), 3 high-intensity interval swims (25–100 m), zone 3 training, and recovery training | 12 hours of training per week (33.4 km), traditional high-volume, low-intensity training | >87% HRmax，RPE≥7 | 60 min | 6-7 times/week | 7 weeks | 50 m performance, 200 m performance, Swimming velocity, Stroke rate, Stroke length |
| Patil et al. (2014) | Core strengthening training: includes a series of exercises such as Prone Plank, Side Plank, Bridging, Bird Dog, Dying Bug, Leg Drop, etc., which gradually increase in difficulty. | Regular swimming training | NA | 30-60 min | 3 times/week | 6 weeks | 50 m performance, Swimming velocity, Stroke rate, Stroke length |
| Potdevin et al. (2011) | Plyometric (Jump) Training: Standing jumps, single leg hops, long jumps, depth jumps, bounding, drop jumps | Regular swimming training | Classification by different height obstacles, platforms and distances | 20-25 min | 2 times/week | 6 weeks | 25 m performance, 50 m performance, Swimming velocity |
| Sadowski et al. (2020) | Power training: The use of dry land water resistance to simulate freestyle stroke movements has strong sport specificity. It uses moderate resistance combined with medium to high frequency strokes to simulate freestyle movements, emphasizes rapid force generation and power conversion, and is more often reflected in explosive power/power training characteristics. | Strength training: The training content used includes: bench press, arm extension, pull-down rowing and other traditional resistance exercises, mainly focusing on high-load resistance training of large muscle groups | 50-60 times/minute | 10 min | 3 times/week | 12 weeks | Swimming velocity, Stroke rate, Stroke length |
| Sammoud et al. (2019) | Plyometric (Jump) Training: twice a week for 8 weeks, mainly includes double-foot jump, CMJ, etc., replacing some special training | Regular swimming training | maximal effort | 25-30 min | 2 times/week | 8 weeks | 25 m performance, 50 m performance |
| Sammoud et al. (2021) | Plyometric (Jump) Training: performed twice a week for 8 weeks, replacing part of the swimming warm-up training. The training included bilateral ankle hops (20cm obstacle) and CMJ. The number of ground contacts per week increased from 50 to 120 times, and the training volume increased week by week. | Regular swimming training | maximal effort | 25-30 min | 2 times/week | 8 weeks | 25 m performance, 50 m performance |
| Sperlich et al. (2010) | HIIT: 5 times a week, each training includes: warm-up (about 400 meters of medley), technical practice (10-15×20 meters of technical interval training), high-intensity interval (30 minutes, intensity is 92% of personal best time), and relaxation. | Regular high volume training | 92% personal best | 30 min | 5 times/week | 5 weeks | 100 m performance |
| Toussaint et al. (1990) | High-resistance training in water was performed using a fixed push-off point (POP) device, three times a week for half an hour each time, with an emphasis on sprint training. | Regular swimming training | maximal effort | 30 min | 8 times/week | 10 weeks | 50 m performance, 100 m performance, 200 m performance, Swimming velocity, Stroke rate |
| Weston et al. (2015) | Core training: including prone-bridge, side-bridge, bird-dog, leg raise, sit twist, shoulder press, etc. | Regular swimming training | The duration and number of sets gradually increased, and the weight of some training sessions was increased to 7kg. | 30 min | 3 times/week | 12 weeks | 50 m performance |
| Jones et al. (2017) | strength training: Heavy weight, slow speed strength training (squat, press, pull, etc.) | power training: High-speed elasticity training (jump squat, box jump, medicine ball throw, etc.) | Strength training: 85–90% 1RM Power training: 80–100% 1RM | 60 min | 3 times/week | 6 weeks | Turn time, Swimming velocity |

Note: N/A, Not Applicable.

# Supplementary 3: Risk of Bias

| **Author** | **Bias arising from the randomization process** | **Bias due to deviations from intended intervention** | **Bias due to missing outcome data** | **Bias in measurement of the outcome** | **Bias in selection of the reported result** | **Overall** |
| --- | --- | --- | --- | --- | --- | --- |
| Amara et al. (2021b) | Low | Low | Low | Low | Low | Low |
| Amara et al. (2022) | Low | Low | Low | Low | Low | Low |
| Born et al. (2020) | Low | Low | Low | Low | Low | Low |
| Amara et al. (2023) | High | Low | High | Low | Low | High |
| Amaro et al. (2017) | Low | Low | Low | Low | Low | Low |
| Aouani et al. (2024) | Low | Low | Low | Low | Low | Low |
| Aspenes et al. (2009) | Low | Low | Low | Some concerns | Low | Some concerns |
| Bishop et al. (2009) | Low | Low | Low | Low | Low | Low |
| Breed et al. (2003) | Low | Low | Low | Low | Low | Low |
| Cañas-Jamett et al. (2020) | Low | Low | Low | Low | Low | Low |
| Chortane et al. (2022) | Low | Low | Low | Some concerns | Low | Some concerns |
| Cossor et al. (1999) | Low | Low | Low | Low | Low | Some concerns |
| Czuba et al. (2017) | Low | Low | Low | Low | Low | Low |
| Dragunas et al. (2012) | Low | Low | Low | Low | Low | Low |
| Garrido et al. (2010) | Low | Low | Low | Low | Low | Low |
| Gencer et al. (2018) | Low | Low | Low | Low | Low | Low |
| Girold et al. (2006) | Low | Low | Low | Low | Low | Low |
| Girold et al. (2007) | Low | Low | Low | Low | Low | Low |
| Girold et al. (2012) | Low | Low | Low | Low | Low | Low |
| Gourgoulis et al. (2019) | Low | Low | Low | Low | Low | Low |
| Karpiński et al. (2020) | Low | Low | Low | Low | Low | Low |
| Khiyami et al. (2022) | Some concerns | Low | Low | Low | Low | Some concerns |
| Kilen et al. (2014) | Low | Low | Low | Low | Low | Low |
| Lopes et al. (2021) | Low | Low | Low | Low | Low | Low |
| Naczk et al. (2016) | Low | Low | Low | Low | Low | Low |
| Norberto et al. (2023) | Low | Low | Low | Low | Low | Low |
| Nugent et al. (2018) | Low | Low | Low | Low | Low | Some concerns |
| Patil et al. (2014) | Low | Low | Low | Low | Low | Low |
| Potdevin et al. (2011) | Low | Low | Low | Low | Low | Low |
| Sadowski et al. (2020) | Low | Low | Low | Low | Low | Low |
| Sammoud et al. (2019) | Low | Low | Low | Low | Low | Low |
| Sammoud et al. (2021) | Low | Low | Low | Low | Low | Low |
| Sperlich et al. (2010) | Low | Low | Low | Low | Low | Low |
| Toussaint et al. (1990) | Low | Low | Low | Low | Low | Low |
| Weston et al. (2015) | Low | Low | Low | Low | Low | Low |
| Jones et al. (2017) | Low | Low | Low | Low | Low | Low |

# Supplementary 4: Funnel plot for the first-level network meta-analysis


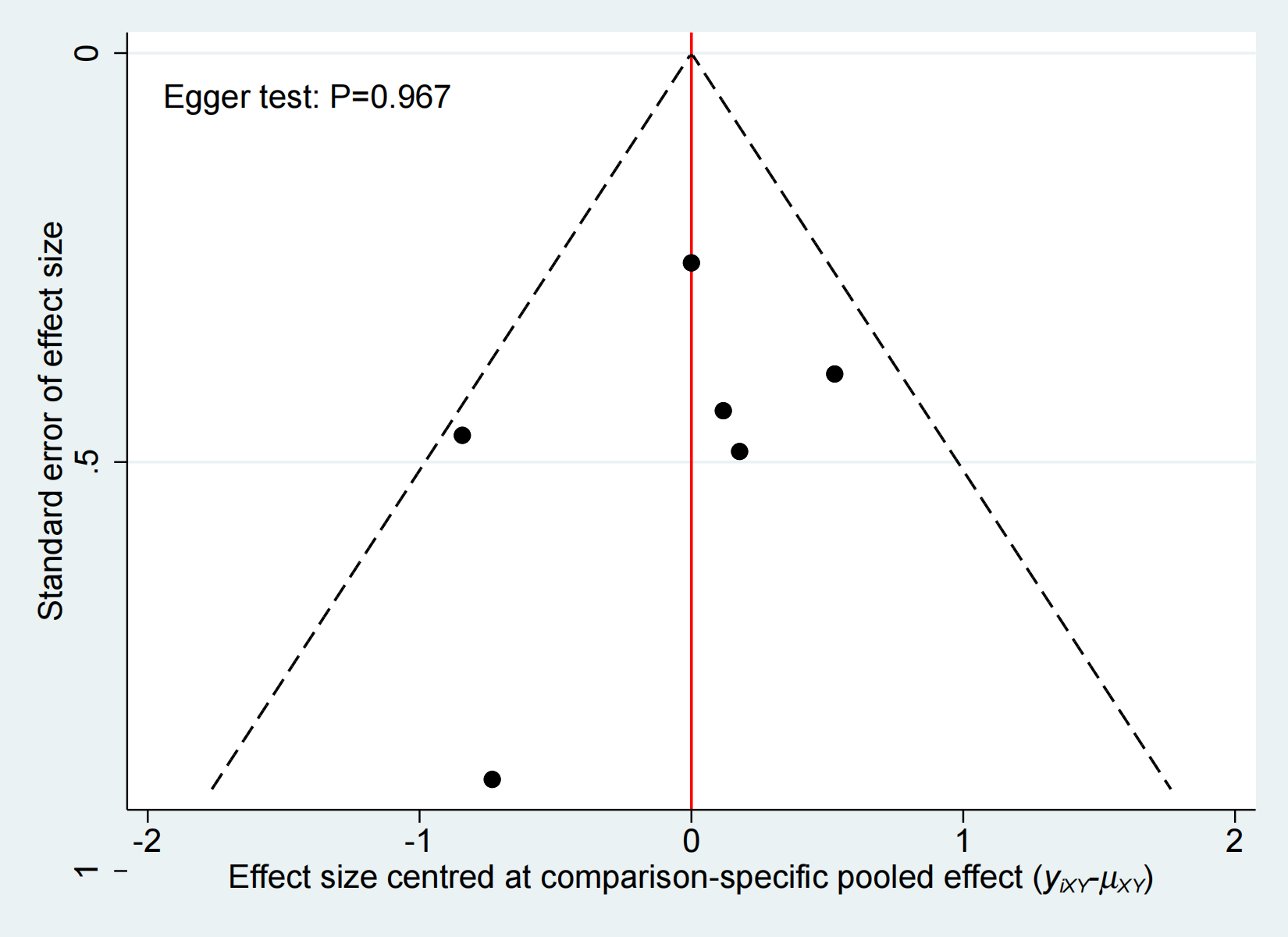


Figure 4.1 The funnel plot for the first-level network meta-analysis of 25m performance. The result of Egger test showed the p=0.967.


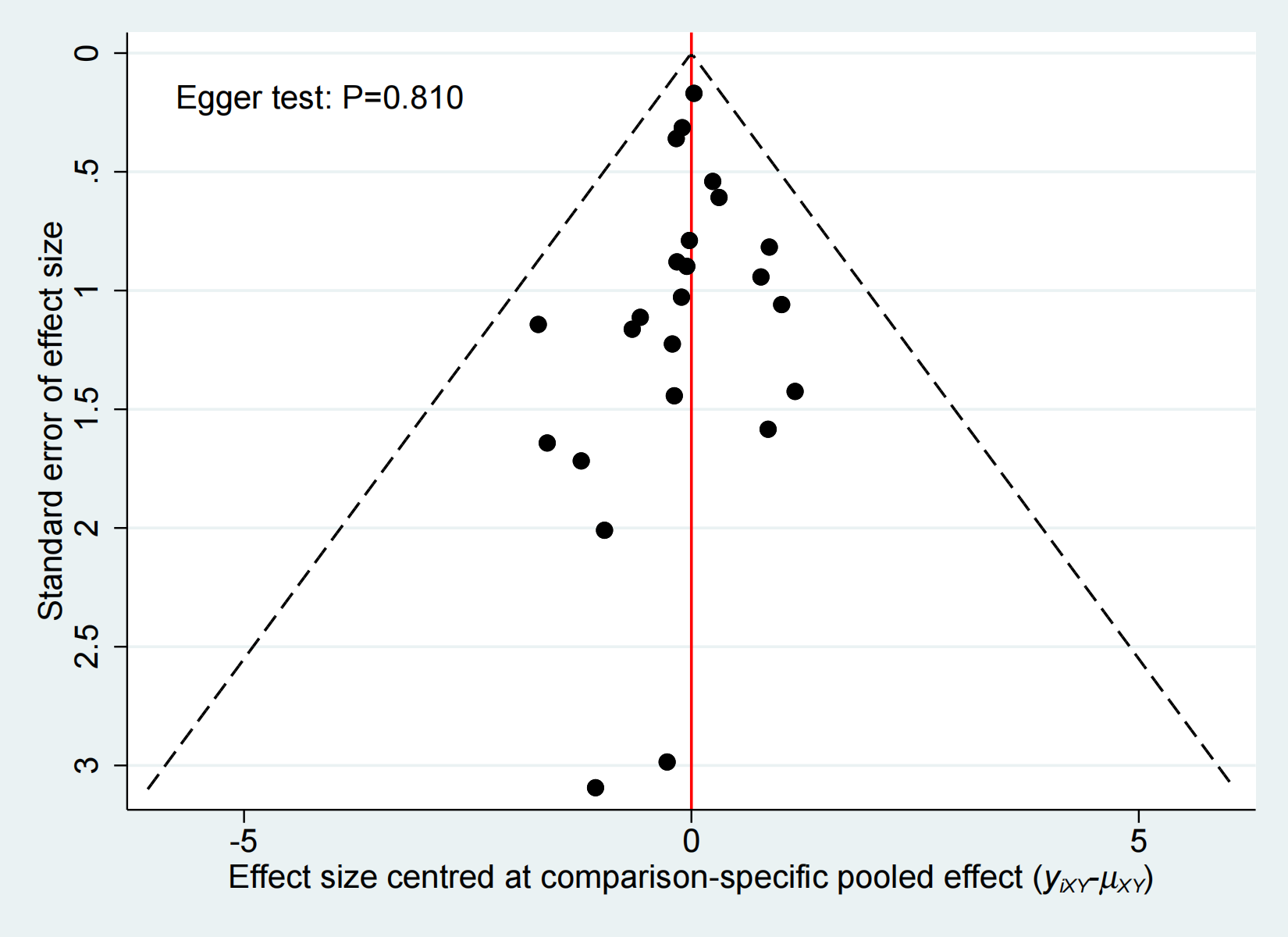


Figure 4.2 The funnel plot for the first-level network meta-analysis of 50m performance. The result of Egger test showed the p=0.810.


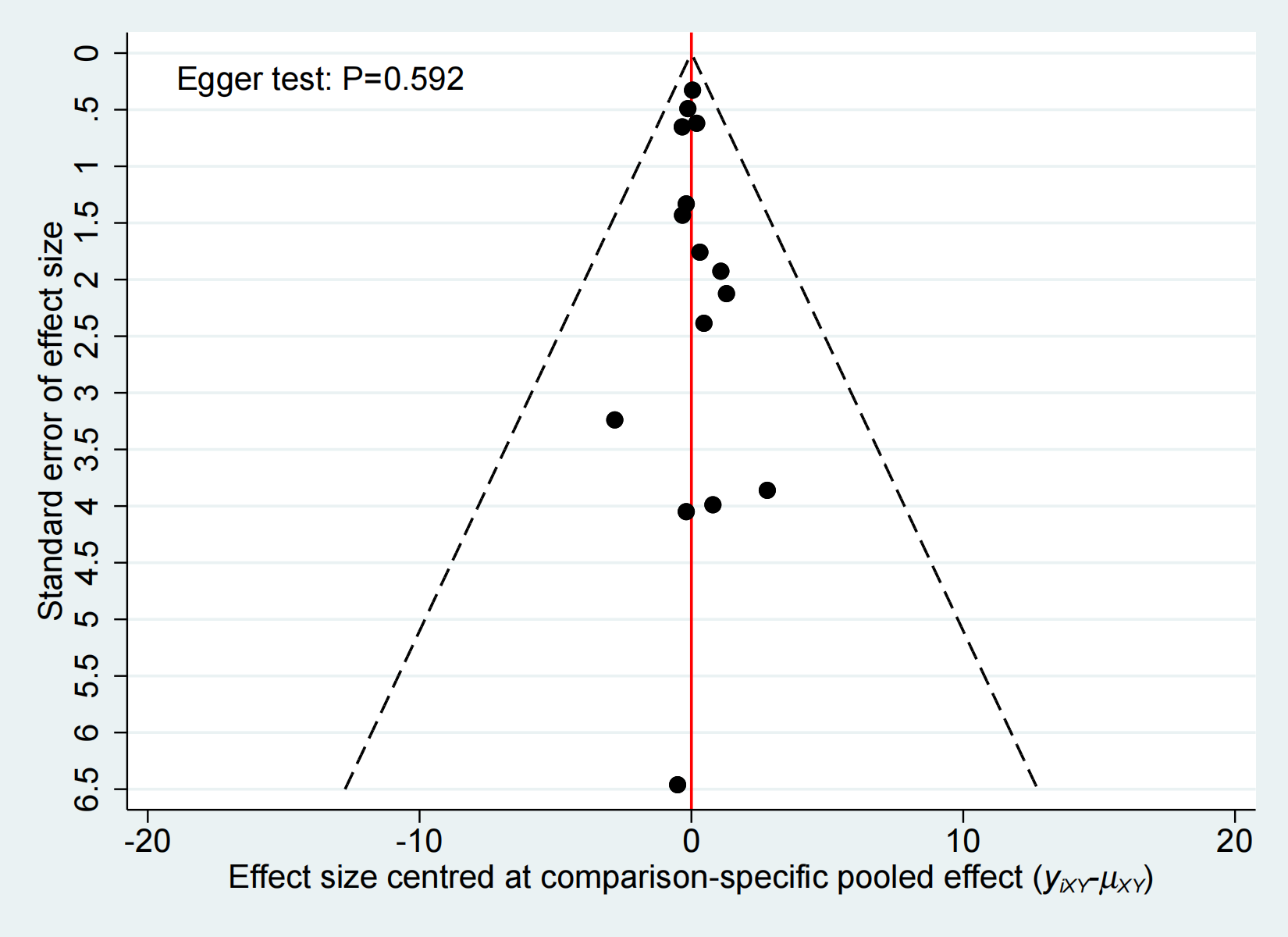


Figure 4.3 The funnel plot for the first-level network meta-analysis of 100m performance. The result of Egger test showed the p=0.592.


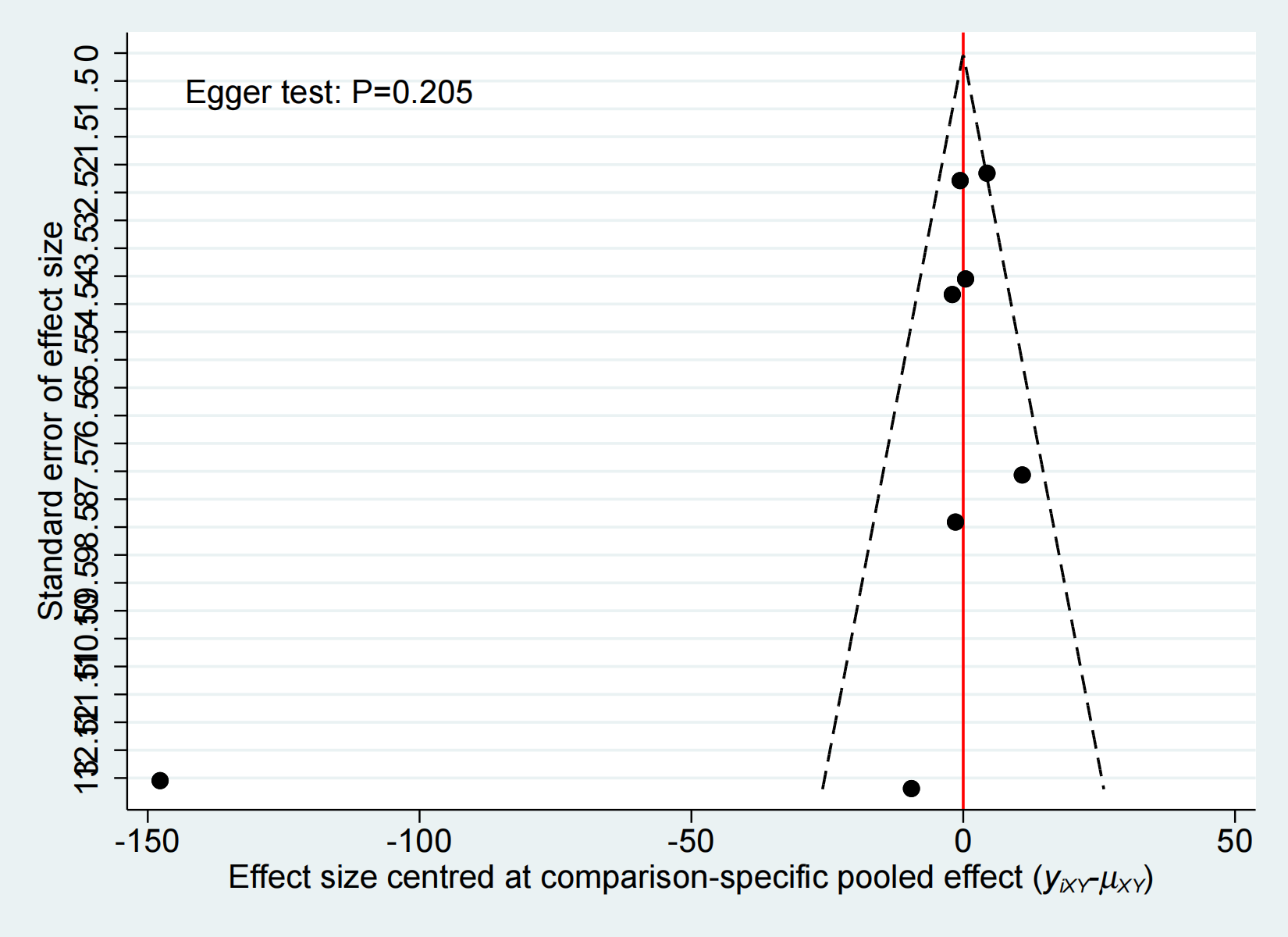


Figure 4.4 The funnel plot for the first-level network meta-analysis of 200m performance. The result of Egger test showed the p=0.205.


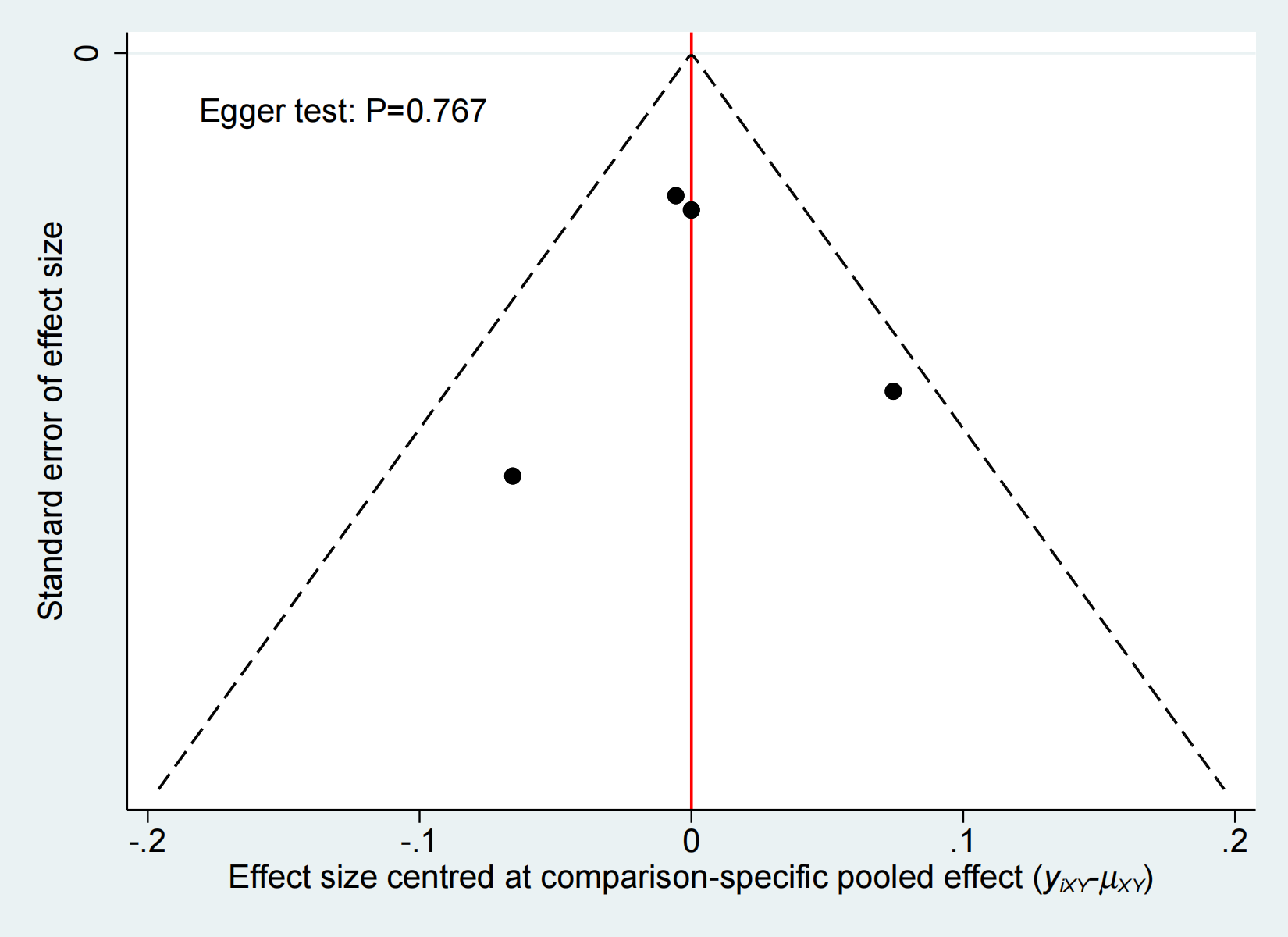


Figure 4.5 The funnel plot for the first-level network meta-analysis of Start time. The result of Egger test showed the p=0.767.


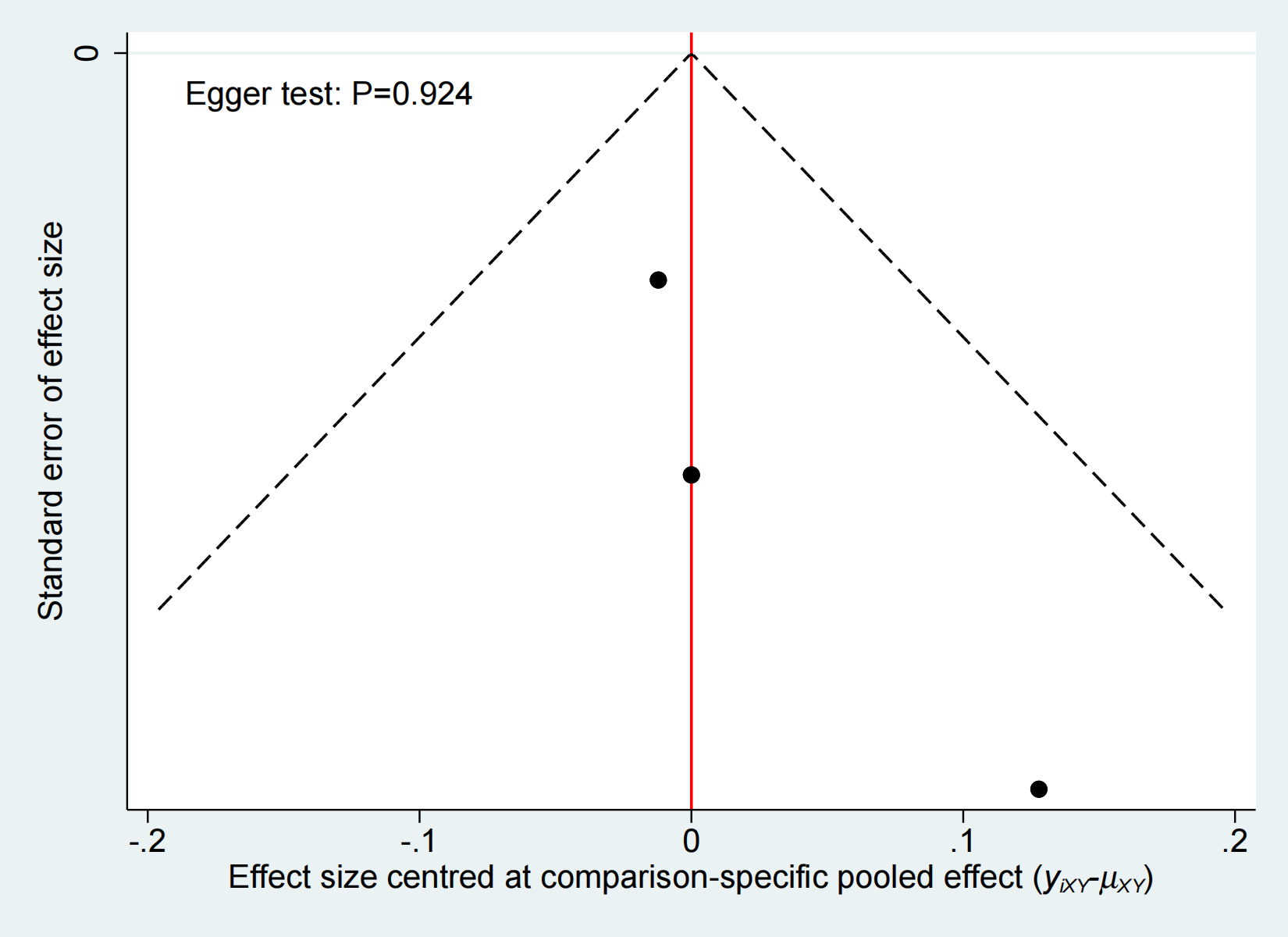


Figure 4.6 The funnel plot for the first-level network meta-analysis of Turn time. The result of Egger test showed the p=0.924.


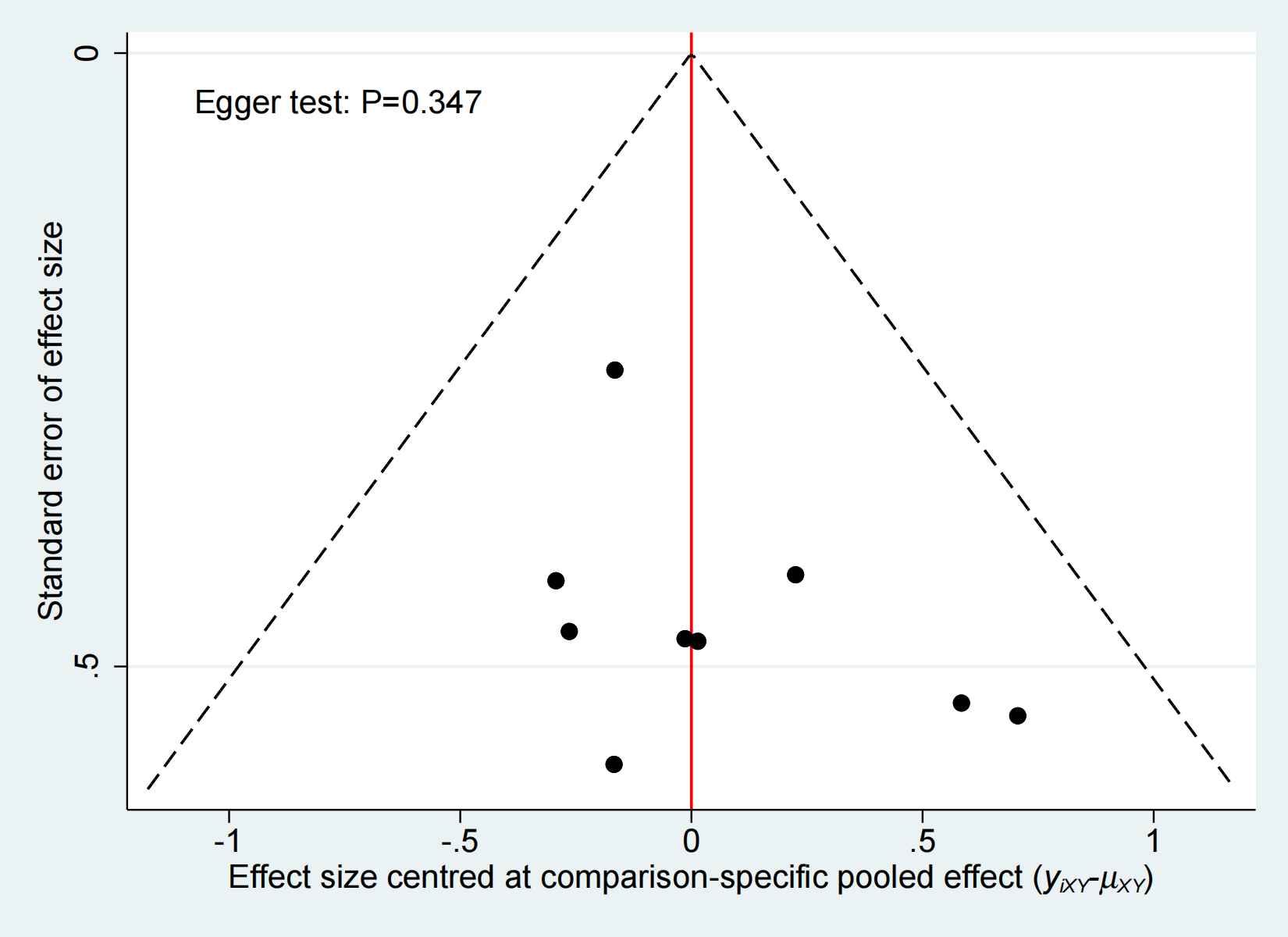


Figure 4.7 The funnel plot for the first-level network meta-analysis of Swimming velocity. The result of Egger test showed the p=0.347.


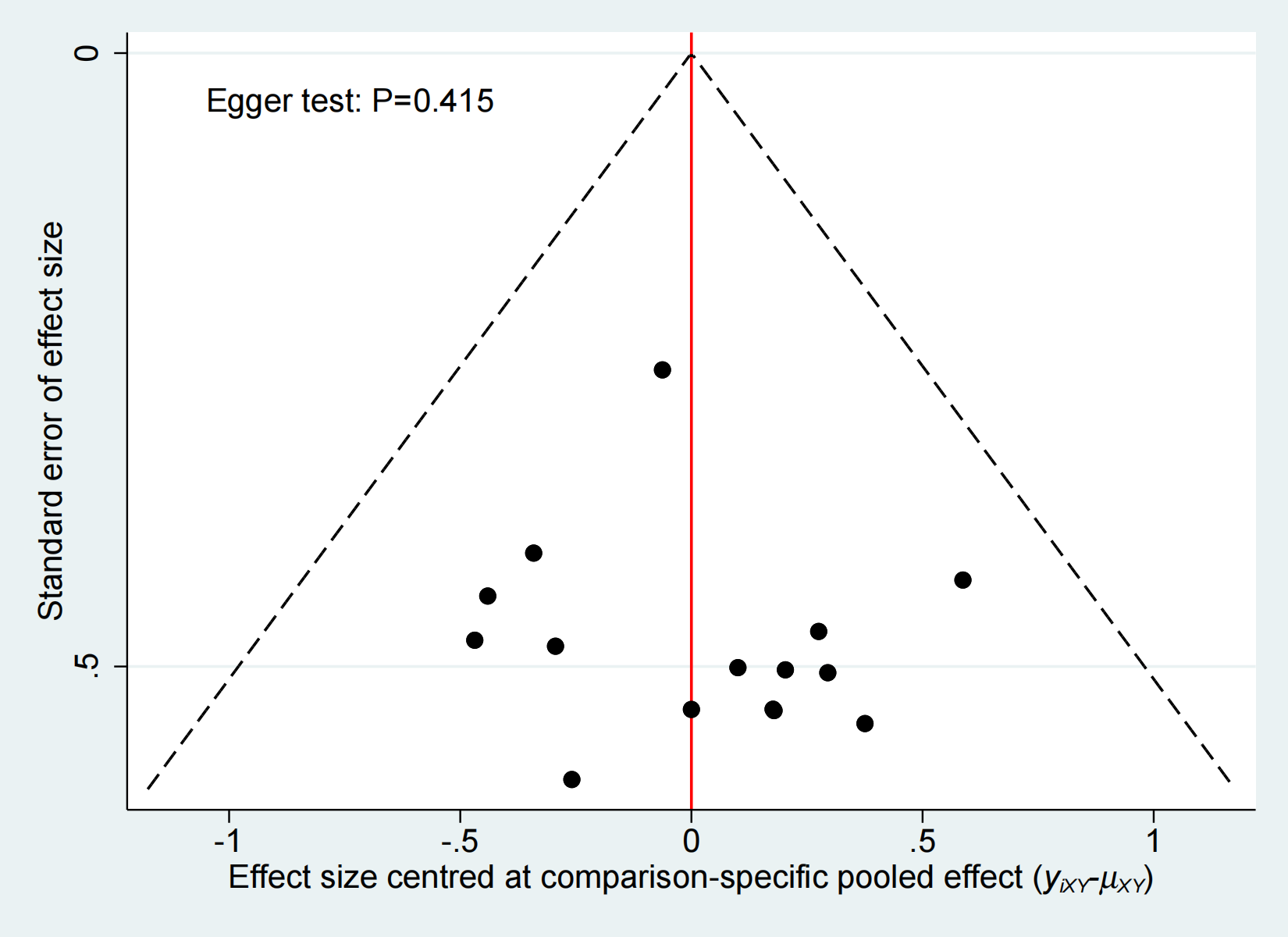


Figure 4.8 The funnel plot for the first-level network meta-analysis of Stroke rate. The result of Egger test showed the p=0.415.


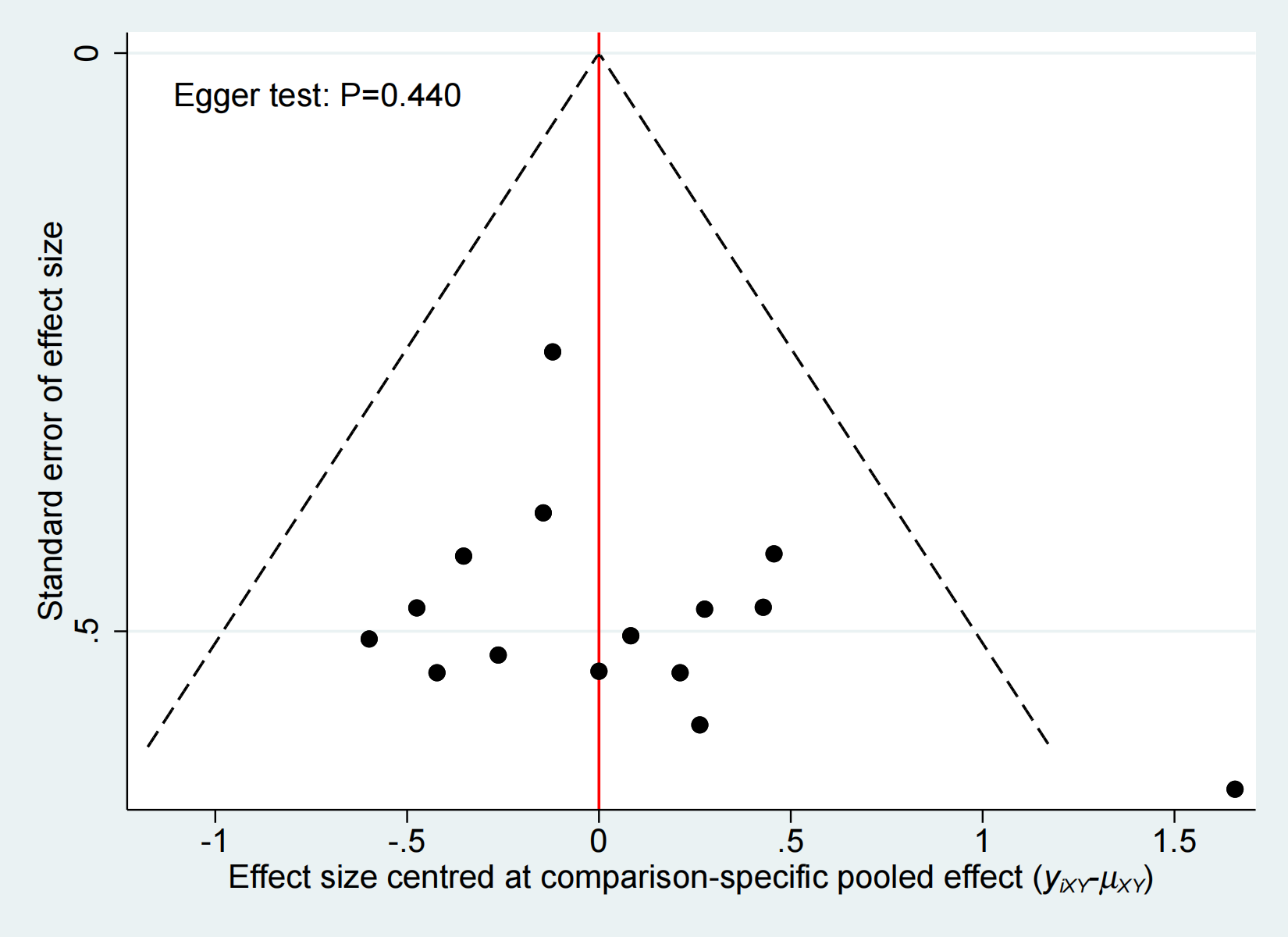


Figure 4.9 The funnel plot for the first-level network meta-analysis of Stroke length. The result of Egger test showed the p=0.440.

# Supplementary 5: Funnel plot for the second-level network meta-analysis


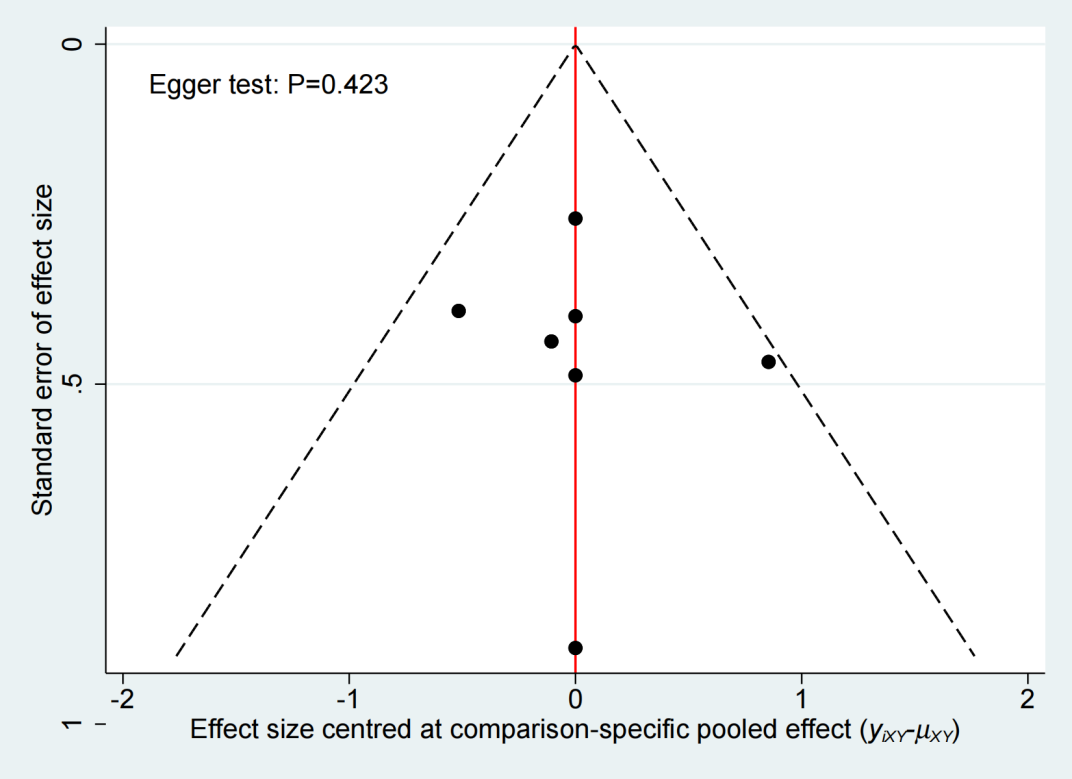


Figure 5.1 The funnel plot for the second-level network meta-analysis of 25m performance. The result of Egger test showed the p=0.423.


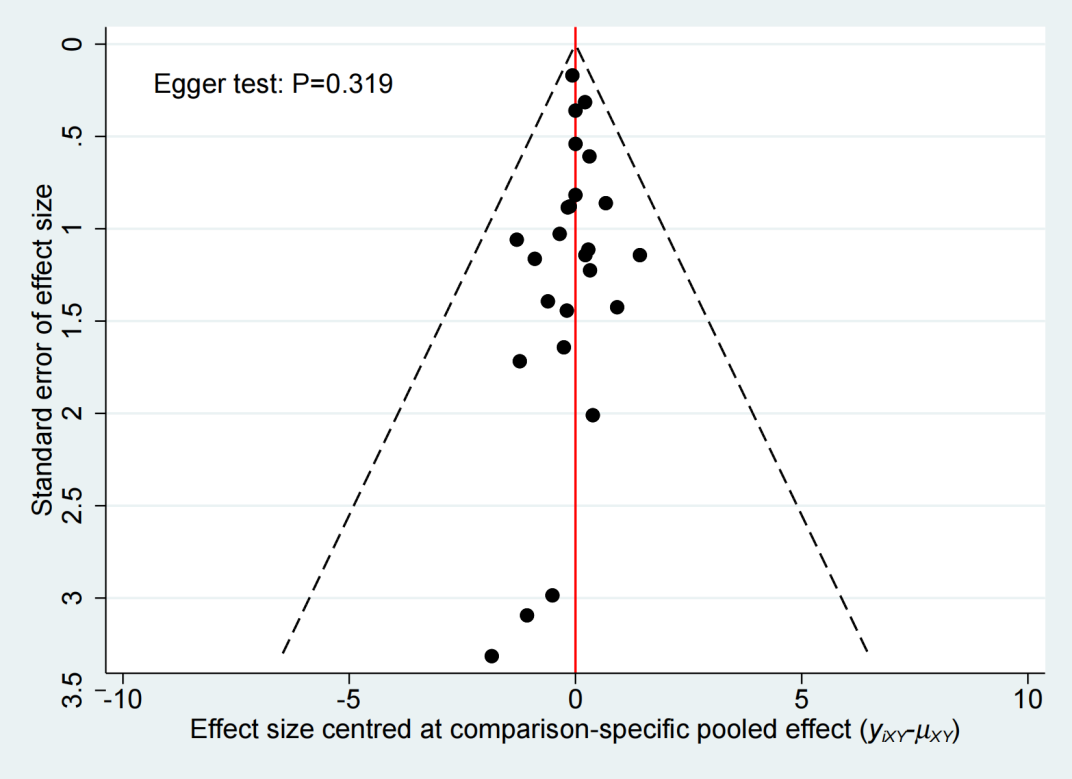


Figure 5.2 The funnel plot for the second-level network meta-analysis of 50m performance. The result of Egger test showed the p=0.319.


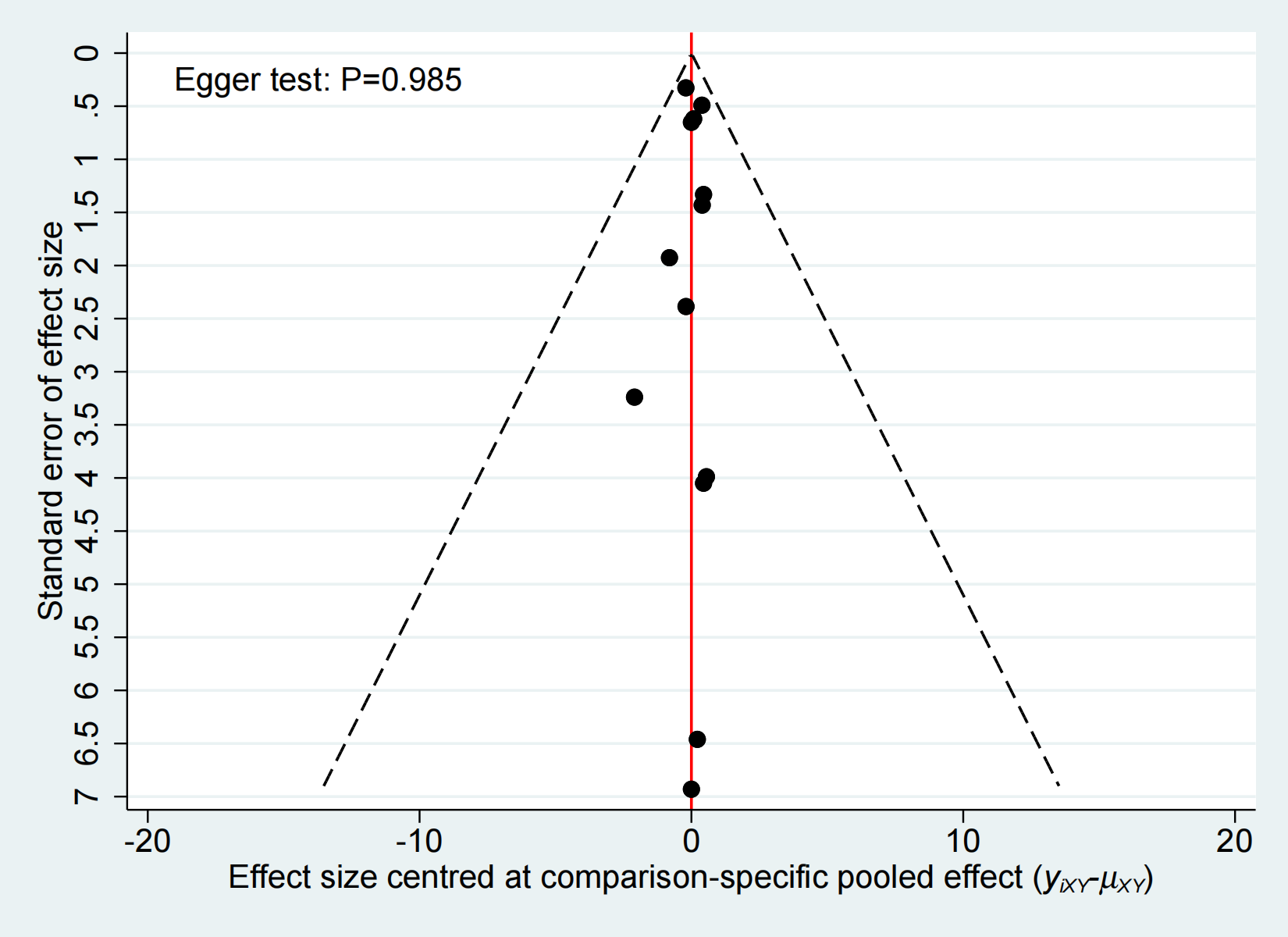


Figure 5.3 The funnel plot for the second-level network meta-analysis of 100m performance. The result of Egger test showed the p=0.985.


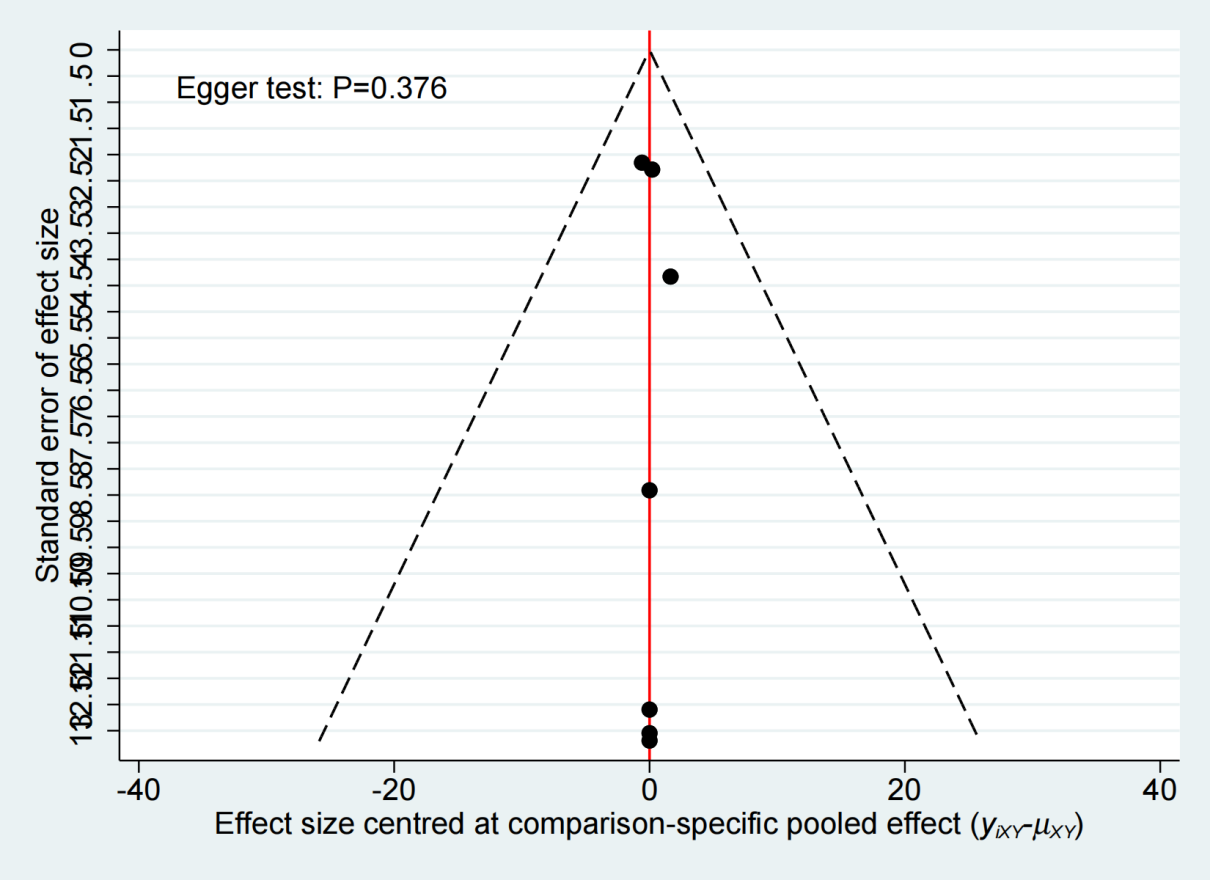


Figure 5.4 The funnel plot for the second-level network meta-analysis of 200m performance. The result of Egger test showed the p=0.376.


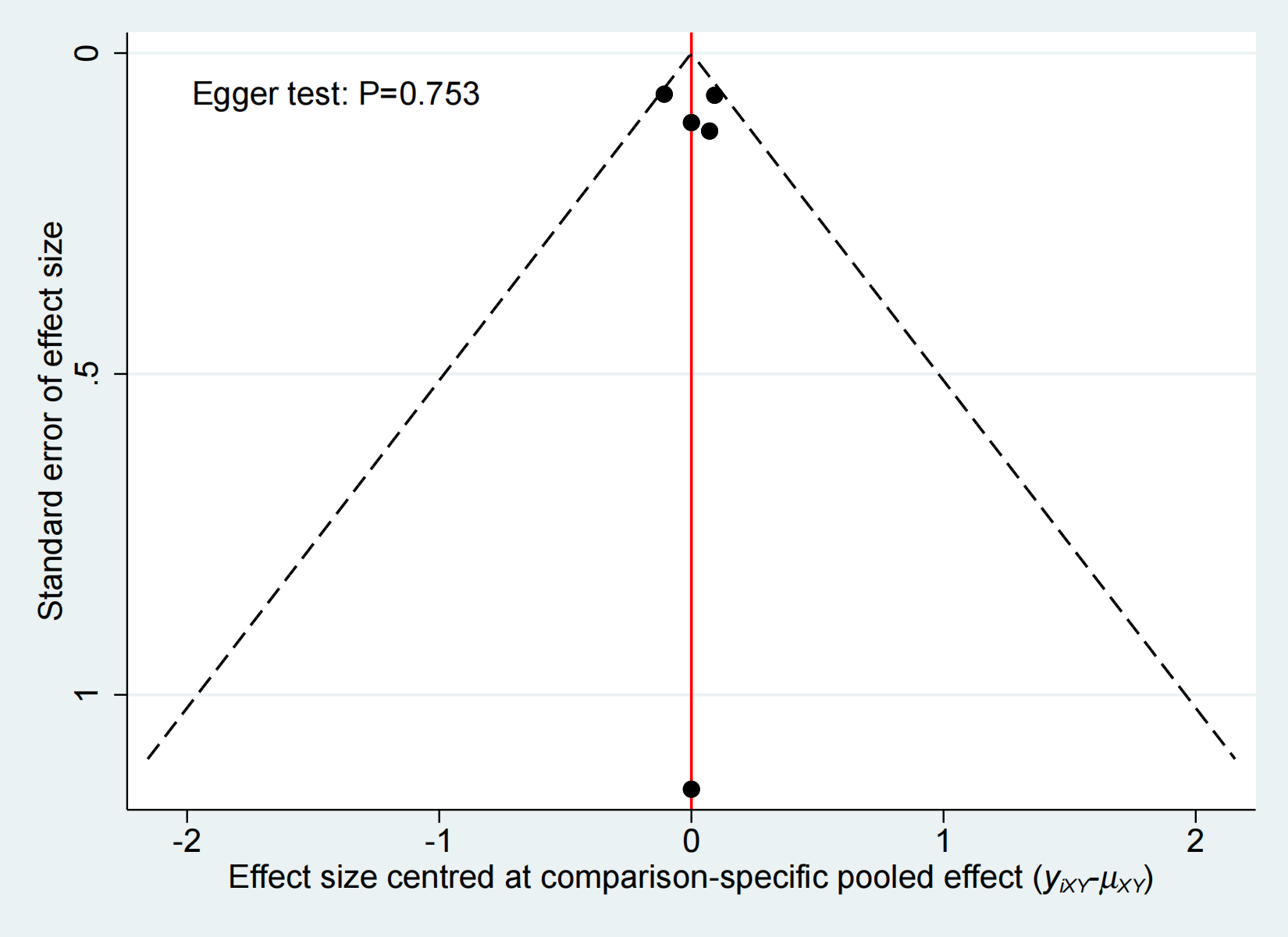


Figure 5.5 The funnel plot for the second-level network meta-analysis of Take-off velocity. The result of Egger test showed the p=0.753.


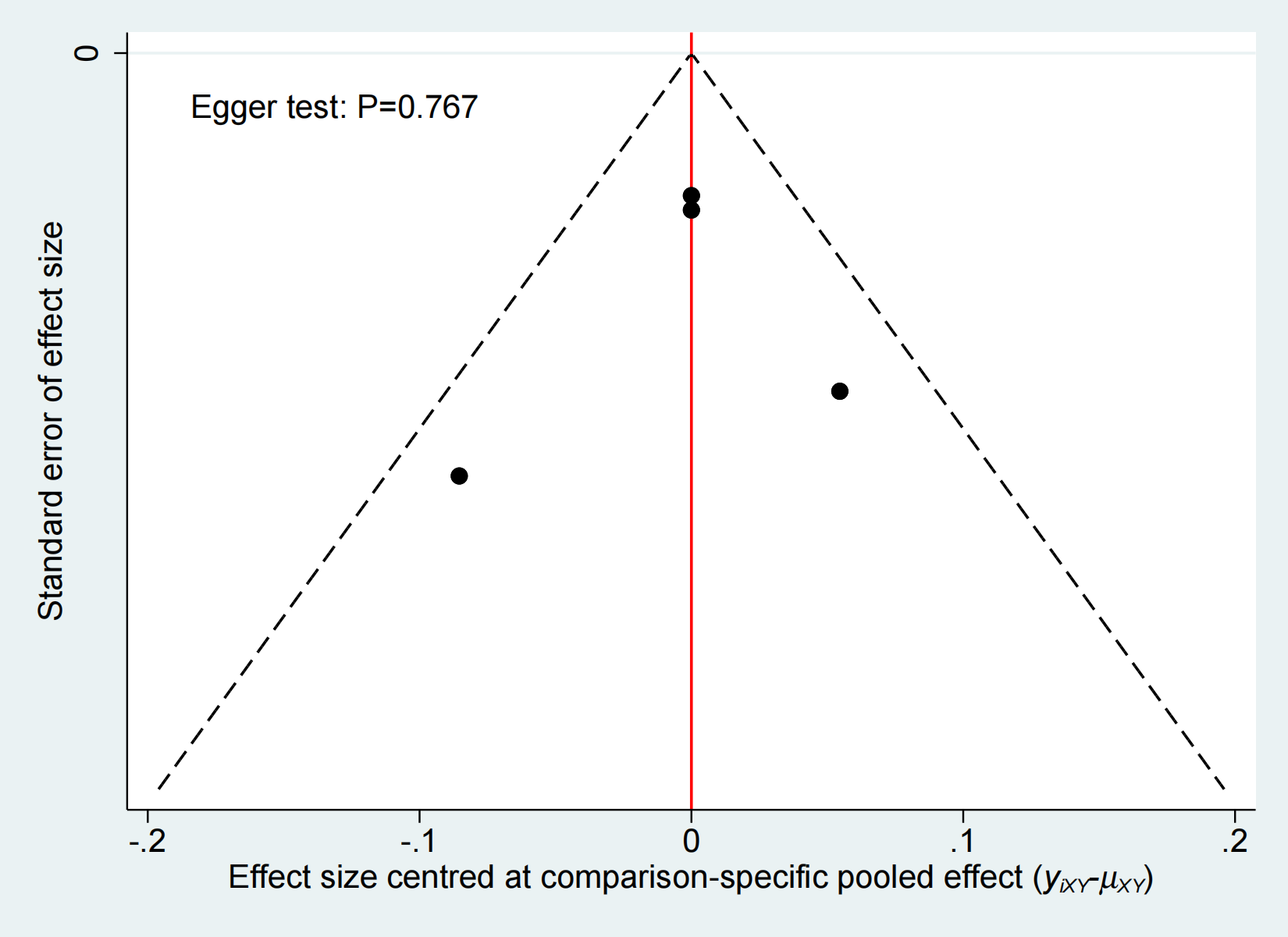


Figure 5.6 The funnel plot for the second-level network meta-analysis of Start time. The result of Egger test showed the p=0.767.


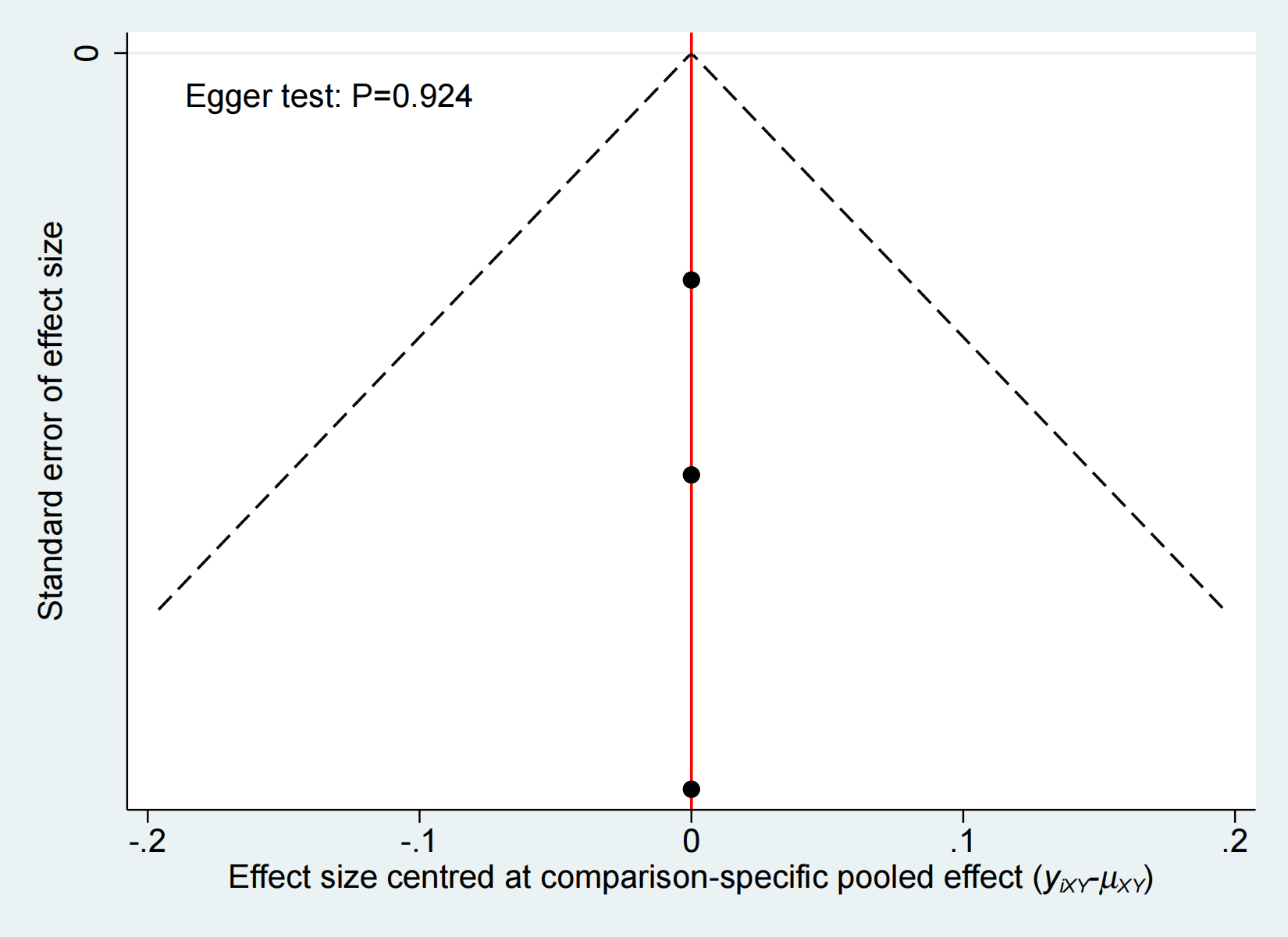


Figure 5.7 The funnel plot for the second-level network meta-analysis of Turn time. The result of Egger test showed the p=0.924.


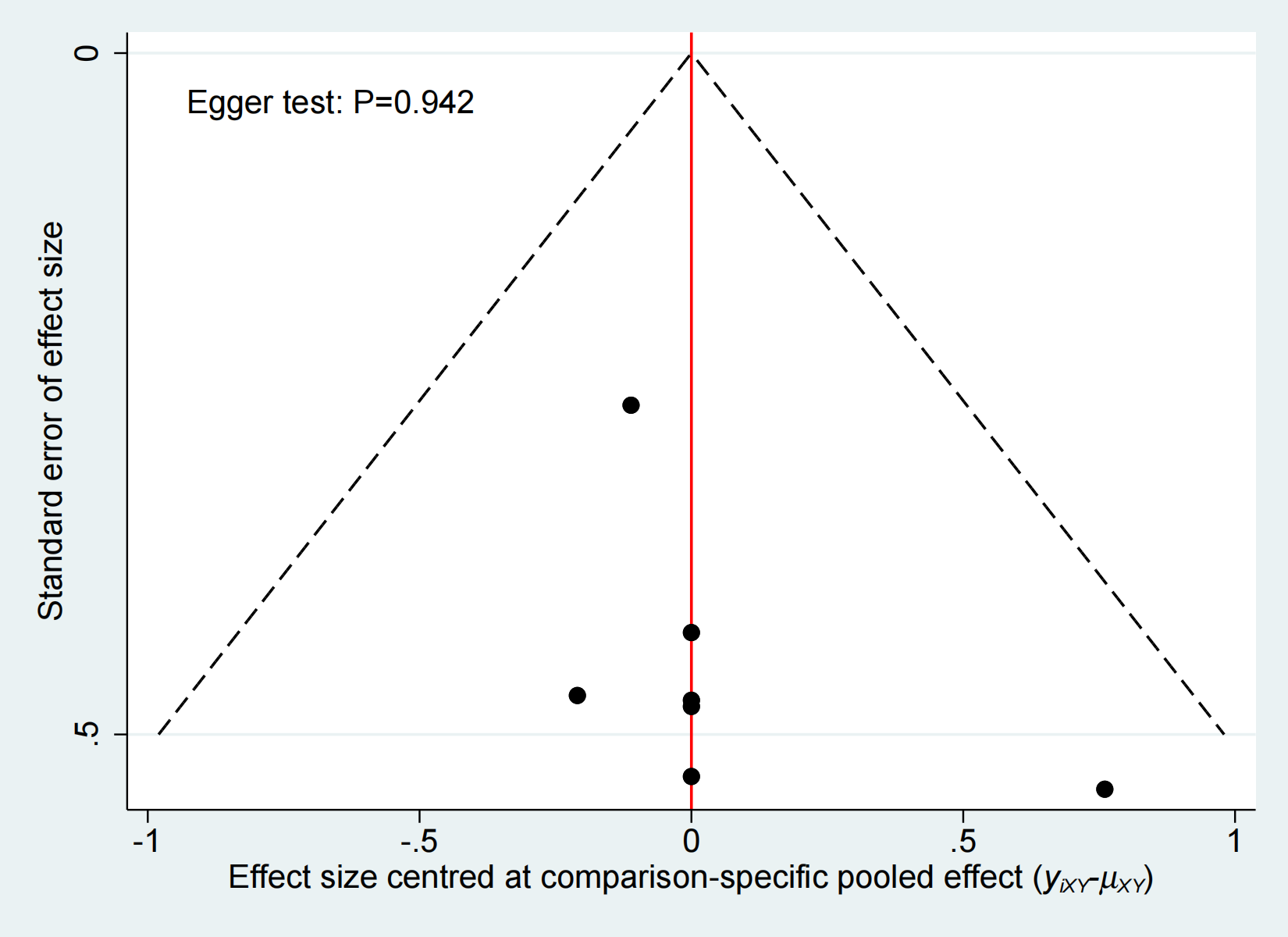


Figure 5.8 The funnel plot for the second-level network meta-analysis of Swimming velocity. The result of Egger test showed the p=0.942.


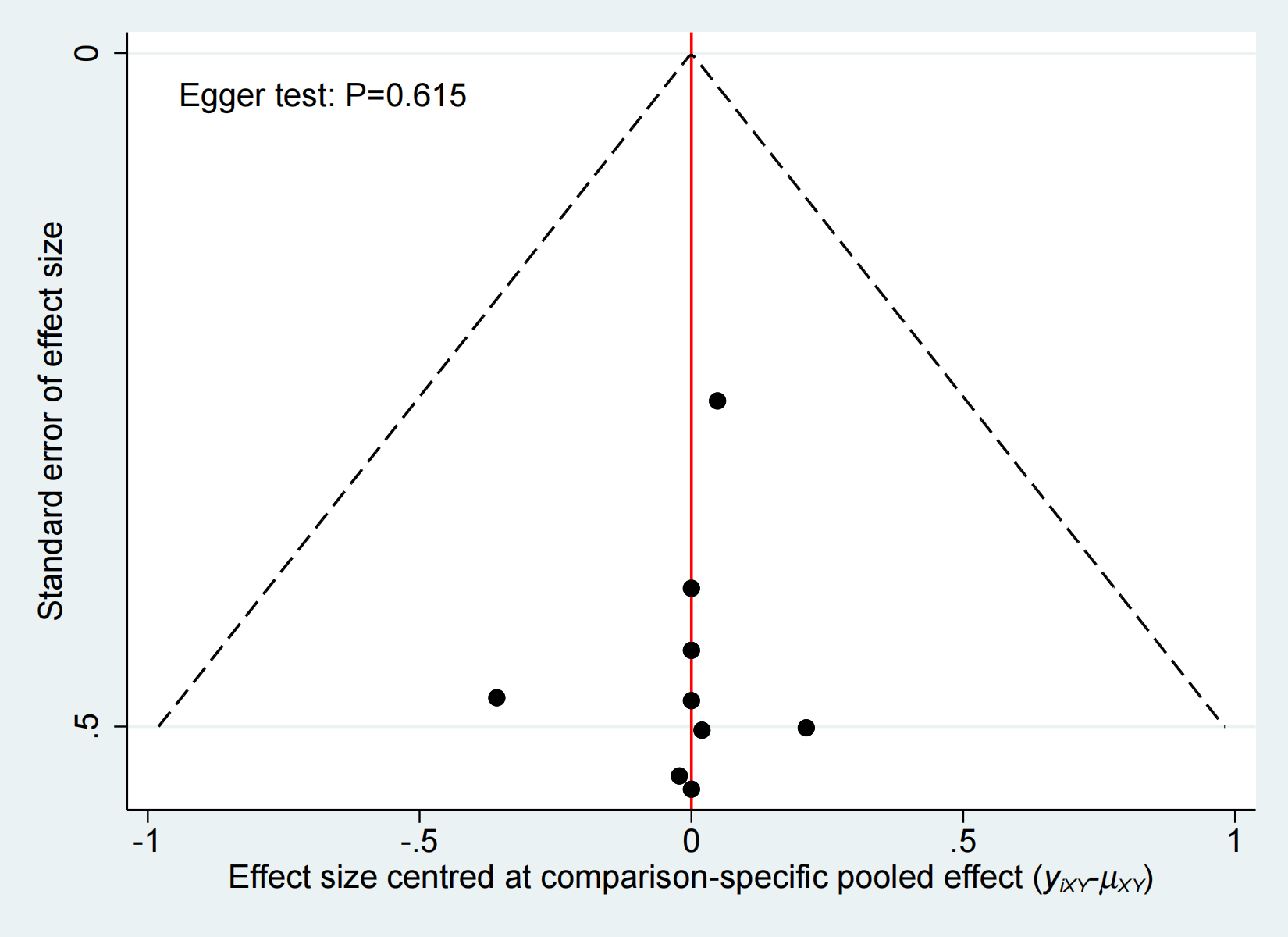


Figure 5.9 The funnel plot for the second-level network meta-analysis of Stroke rate. The result of Egger test showed the p=0.615.


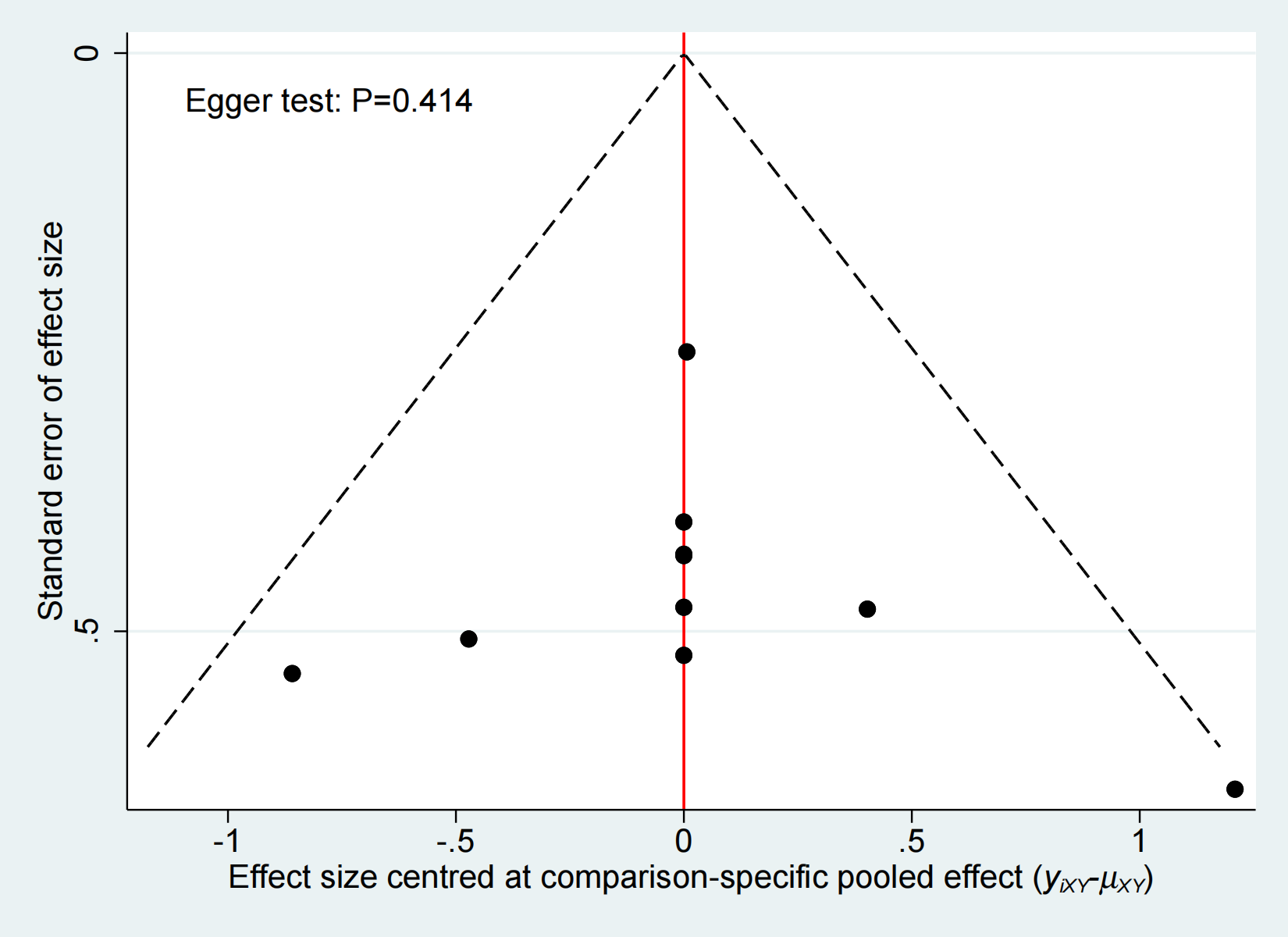


Figure 5.10 The funnel plot for the second-level network meta-analysis of Stroke length. The result of Egger test showed the p=0.414.
